# Microbial and human transcriptome in vaginal fluid at midgestation: Association with spontaneous preterm delivery

**DOI:** 10.1002/ctm2.1023

**Published:** 2022-09-14

**Authors:** Tove Wikström, Sanna Abrahamsson, Johan Bengtsson‐Palme, Joakim Ek, Pihla Kuusela, Elham Rekabdar, Peter Lindgren, Ulla‐Britt Wennerholm, Bo Jacobsson, Lil Valentin, Henrik Hagberg

**Affiliations:** ^1^ Centre of Perinatal Medicine and Health Department of Obstetrics and Gynecology Institute of Clinical Sciences Sahlgrenska Academy University of Gothenburg Gothenburg Sweden; ^2^ Department of Obstetrics Region Västra Götaland Sahlgrenska University Hospital Gothenburg Sweden; ^3^ Bioinformatics Core Facility Sahlgrenska Academy University of Gothenburg Gothenburg Sweden; ^4^ Department of Infectious Diseases Institute of Biomedicine Sahlgrenska Academy University of Gothenburg Gothenburg Sweden; ^5^ Centre for Antibiotic Resistance Research (CARe) at University of Gothenburg Gothenburg Sweden; ^6^ Division of Systems and Synthetic Biology Department of Biology and Biological Engineering Chalmers University of Technology Gothenburg Sweden; ^7^ Institute of Neuroscience and Physiology Department of Physiology Sahlgrenska Academy University of Gothenburg Gothenburg Sweden; ^8^ Södra Älvsborg Hospital Borås Sweden; ^9^ Department of Clinical Science Intervention and Technology Karolinska Institutet Stockholm Sweden; ^10^ Centre for Fetal Medicine Karolinska University Hospital Stockholm Sweden; ^11^ Department of Obstetrics and Gynecology Skåne University Hospital Malmö Sweden; ^12^ Department of Clinical Sciences Malmö Lund University Lund Sweden

**Keywords:** gene expression profiles, human microbiome, infection, microbial community composition, pregnancy, preterm birth, transcriptome, vagina

## Abstract

**Background:**

Intrauterine infection and inflammation caused by microbial transfer from the vagina are believed to be important factors causing spontaneous preterm delivery (PTD). Multiple studies have examined the relationship between the cervicovaginal microbiome and spontaneous PTD with divergent results. Most studies have applied a DNA‐based assessment, providing information on the microbial composition but not transcriptional activity. A transcriptomic approach was applied to investigate differences in the active vaginal microbiome and human transcriptome at midgestation between women delivering spontaneously preterm versus those delivering at term.

**Methods:**

Vaginal swabs were collected in women with a singleton pregnancy at 18 + 0 to 20 + 6 gestational weeks. For each case of spontaneous PTD (delivery <37 + 0 weeks) two term controls were randomized (39 + 0 to 40 + 6 weeks). Vaginal specimens were subject to sequencing of both human and microbial RNA. Microbial reads were taxonomically classified using Kraken2 and RefSeq as a reference. Statistical analyses were performed using DESeq2. GSEA and HUMAnN3 were used for pathway analyses.

**Results:**

We found 17 human genes to be differentially expressed (false discovery rate, FDR < 0.05) in the preterm group (*n* = 48) compared to the term group (*n* = 96). Gene expression of kallikrein‐2 (KLK2), KLK3 and four isoforms of metallothioneins 1 (MT1s) was higher in the preterm group (FDR < 0.05). We found 11 individual bacterial species to be differentially expressed (FDR < 0.05), most with a low occurrence. No statistically significant differences in bacterial load, diversity or microbial community state types were found between the groups.

**Conclusions:**

In our mainly white population, primarily bacterial species of low occurrence were differentially expressed at midgestation in women who delivered preterm versus at term. However, the expression of specific human transcripts including KLK2, KLK3 and several isoforms of MT1s was higher in preterm cases. This is of interest, because these genes may be involved in critical inflammatory pathways associated with spontaneous PTD.

## Graphical abstract

Kallikreins (KLKs) and metallothioneins (MTs) mRNA are increased in vaginal fluid in gestational week 18‐20 in women who subsequently deliver preterm. MTs are activated in response to cytokine‐mediated inflammation and oxidative stress (1). MTs regulate metalloproteinases which degrades the extracellular matrix and have been associated with preterm prelabour rupture of foetal membranes (PPROM) (2). KLKs can be mechanistically involved in preterm delivery via degradation of the cervical mucus barrier (3), modulation of anti‐microbial peptides (4) and preterm prelabour rupture of foetal membranes (PPROM) (5).

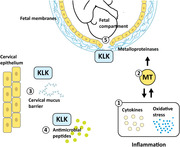

## 1 INTRODUCTION

Preterm delivery (PTD), defined as delivery before 37 gestational weeks,^1^ is a health problem worldwide. Globally 15 million babies are estimated to be born too early, with an occurrence ranging from 5% of all births in northern Europe to 18% in some African countries.^2^, ^3^ PTD accounts for a substantial proportion of perinatal mortality and neonatal morbidity^4^, ^5^ causing a considerable burden on families and caregivers as well as on health and social services.^6^

PTD is commonly divided into spontaneous PTD and indicated PTD because of the difference in underlying pathophysiology and clinical presentation. The former includes spontaneous onset of labour and preterm prelabour rupture of membranes, whereas the latter is explained by caesarean delivery or induction of labour because of maternal or foetal complications.^7^ Spontaneous PTD is of particular interest, since a significant proportion of spontaneous PTDs occurs at extremely low gestational age, and mortality and morbidity are high in this subgroup of preterm infants.^8^

The pathogenesis of spontaneous PTD is still poorly understood despite immense investigational efforts.^8^, ^9^ Several studies have shown that a short cervix as measured with transvaginal ultrasound in the second trimester increases the risk of spontaneous PTD, even though the discriminative ability of cervical length is at best moderate.^10^, ^11^, ^12^, ^13^, ^14^ Although a sonographically short cervix is a reasonably good predictor of spontaneous PTD, it is a surrogate marker for the premature remodelling of the cervix. A short cervix is only one crucial component in the parturition process.^15^ To sharpen the diagnostics and to identify novel prophylactic interventions, it is essential to understand the underlying molecular events causing spontaneous PTD.

In recent years the interaction between the cervicovaginal microbiome and its host has been suggested to play an important role in health and disease development in both women of reproductive age and postmenopausal women.^16^, ^17^, ^18^, ^19^, ^20^, ^21^, ^22^ The general perception is that “normal” cervicovaginal microbiota in women of reproductive age is dominated by *Lactobacillus* species. In contrast, an abnormal microbiota is characterized by low abundance of lactobacilli with an overgrowth of anaerobic bacteria, such as *Gardernella vaginalis*, *Prevotella* spp., *Bacteroides* spp. and Mollicutes.^23^ Multiple studies have examined the relationship between the cervicovaginal microbiome and spontaneous PTD with divergent results.^24^, ^25^, ^26^, ^27^, ^28^, ^29^, ^30^, ^31^, ^32^, ^33^, ^34^, ^35^ The differing results are probably explained by differences in race or ethnicity between the study populations,^26^, ^32^, ^33^ differences in gestational age at sampling, cervicovaginal sampling procedure and the analytic methodological strategy. Most studies, but not all,^31^ that have examined the association between the cervicovaginal microbiome and spontaneous PTD have applied 16s rRNA gene sequencing. Studies using a DNA‐based analysis provide information on the taxonomic composition of the bacterial communities but not on whether the bacteria are active members of the microbiome.^36^ Furthermore, studies based on the 16s rRNA gene have limited taxonomic resolution at the species level, compared to studies sampling reads from the entire genome or transcriptome of the studied species.^37^ In the present study we performed RNA sequencing by using high throughput sequencing technology to quantify the transcriptome, that is, the transcriptionally active part of the genome.^38^ The aim was to assess whether there are differences in the active vaginal microbiome (metatranscriptome) and human transcriptome in the second trimester between women who subsequently deliver spontaneously preterm and those who deliver spontaneously at term.

## 2 METHOD

### 2.1 Study design

The study is a nested case‐control study within the CERVIX‐study, a prospective blinded multicentre study conducted at six university hospitals and one regional hospital in Sweden, including 11 072 women with transvaginal cervical length measurement at 18 + 0 to 20 + 6 weeks and delivery data. (https://doi.org/10.1186/ISRCTN18093885). The CERVIX‐study has been described in detail elsewhere.^14^ In short, asymptomatic women (i.e., without signs of preterm labour) with a singleton pregnancy were consecutively recruited to the CERVIX‐study at their routine second trimester foetal ultrasound examination, which included foetal biometry for estimation of gestational age and foetal anatomy scanning. Those ≥18 years old with a live singleton pregnancy between 18 gestational weeks + 0 days (18 + 0 weeks) and 20 + 6 gestational weeks were invited to participate. Gestational age was estimated on the basis of ultrasound measurement of the foetal biparietal diameter,^39^, ^40^ or on the day of embryo transfer in case of in vitro fertilization according to Swedish guidelines (https://www.sfog.se/media/336451/fetometri.pdf). Women with foetal malformations detected at the ultrasound scan, ruptured membranes, symptoms or findings indicating ongoing miscarriage, current use of progesterone or cerclage in situ, difficulties with understanding written or oral information about the study, and women with missing information about pregnancy outcome were excluded.

Between March 2016 and June 2017, women who accepted to participate in the CERVIX‐study at Sahlgrenska University Hospital, Skåne University Hospital and Karolinska University Hospital were also asked to agree to sampling of vaginal fluid. The study protocol included collection of vaginal fluid from the posterior vaginal fornix prior to transvaginal ultrasound measurement of cervical length at 18 + 0 to 20 + 6 gestational weeks. Women who had had vaginal intercourse within the past 48 h were not sampled.

### 2.2 Biospecimen collection

The biospecimen collection was done by the specially trained midwife sonographers who performed the cervical length measurements in the CERVIX‐study.^14^ A standardized collection technique was used. A speculum was introduced into the vagina to visualize the cervix and the posterior fornix. After visualization of the cervix and posterior fornix, a cotton swab was rolled in the posterior fornix. All swab samples were placed in QIAzol lysis reagent (Qiagen, Valencia, CA) to prevent RNA degradation. The samples were stored at minus 80°C within 2 h of collection until later use. We have confirmed (unpublished results) that this technique provides adequate conservation of mRNA for expression analysis.

## 3 REFERENCE STANDARD AND GROUP COMPOSITION

Reference standard was set to gestational age at delivery based on foetal biometry (see above), with the primary outcome of spontaneous PTD < 37 + 0 gestational weeks including late spontaneous miscarriage. We define late miscarriage as delivery before 22 + 0 gestational weeks of a foetus showing no signs of life.

The diagnosis of spontaneous PTD and spontaneous late miscarriage in the index pregnancy was validated by scrutiny of medical records. Spontaneous PTD was defined as delivery <37 + 0 gestational weeks (<259 days), either after spontaneous onset of labour or after preterm prelabour rupture of membranes, the latter regardless of whether labour was induced or not.

For each case of spontaneous PTD or late miscarriage two controls were selected. The controls were chosen using simple random selection of women who had completed the study with spontaneous start of delivery at 39 + 0 to 40 + 6 gestational weeks, either after spontaneous onset of labour or after prelabour rupture of membranes. The interval 39 + 0 to 40 + 6 weeks was chosen to obtain a control group that was as normal as possible, considering that both delivery at early term and late term or post term is associated with a slightly higher risk of complications than delivery at midterm.^41^, ^42^, ^43^, ^44^, ^45^

### 3.1 Clinical data collection

Information on the participants’ self‐reported ethnicity was retrieved from the electronic case record form (standardized anamnestic information obtained from the women at enrolment). Other information on participant characteristics and pregnancy outcome was obtained from the Swedish Pregnancy Register (www.graviditetsregistret.se).^46^ If delivery data was missing in the Swedish Pregnancy Register, medical records were scrutinized. If no information was found in the records, the participants were contacted by mail or telephone.

Information on redeemed prescription of vaginal progesterone after registration in the study was retrieved from the Swedish Prescribed Drug Register. The Swedish National Patient Register was used to obtain information on cerclage insertion after inclusion and on cervical conization before inclusion in the study. Information on previous spontaneous PTD (singleton or multiple) was obtained from the Swedish Medical Birth Register, and in case of ambiguous information regarding type of previous PTD, the medical records were searched. The Swedish Prescribed Drug Register, the Swedish National Patient Register, and the Swedish Medical Birth Register are hosted by the Swedish National Board of Health and Welfare (www.socialstyrelsen.se).

### 3.2 Statistical analysis of clinical data

Results for continuous variables are presented as mean and standard deviation, or as median, interquartile range, minimum and maximum. Results for categorial variables are presented as numbers and percentages. For comparison between groups Fisher´s Exact test (2 sided) was used for dichotomous variables, Mantel–Haenszel Chi Square test was used for ordered categorical variables, Chi Square test was used for non‐ordered categorical variables and *t*‐test was used for continuous variables. The Statistical software SAS System Version 9.4, SAS‐Institute, Cary, NC, USA was used for these analyses.

### 3.3 Total RNA isolation

Total RNA including mRNA, miRNA and other small RNA were extracted from the vaginal fluid specimens by using phenol/guanidine‐base lysis of samples. After addition of chloroform, silica‐membrane‐based purification was performed as described in the manufacture's protocol for Qiagen´s miRNeasy Micro Kit. The RNA was eluted in 20 μl of RNase‐free water and the concentration was determined with NanoDrop 2000™ (Thermo Scientific™, Waltham, MA). The RNA integrity number (RIN) was verified by using the Tapestation 2200 RNA screenTape (Agilent).

### 3.4 High throughput sequencing

The Illumina TruSeq Total Stranded RNA kit with RiboZero (Gold) Sample Preparation Guide (15031048 Rev. E) was used. The samples were divided in three groups depending on their RIN‐value, followed by optimization of the fragmentation time of the mRNA depending on the RIN‐value as described below.

A total of 10 μl (∼1 μg) from each sample was used for starting the library preparation. Directly after depletion, a clean‐up was performed by using 200 μl of the RNAClean XP beads (Beckman Coulter, USA) for each sample. The fragmentation step was performed with the following modifications: RNA samples with a RIN‐value of 1‐4 were fragmented for 3 min, samples with a RIN‐value of 5‐6 for 6 min, and samples with a RIN‐value of 7‐10 for 8 min. The PCR cycle was set to 13 cycles for all samples.

Libraries were quantified and normalized using the Qubit DNA HS Assay kit (Life Technologies) and Tapestation 2200 (Agilent), as recommended by Illumina. The libraries were prepared for pooling using the Illumina protocol (Index Adapters Pooling Guide V05) and were sequenced on a NovaSeq 6000 S1 reagent 200 C for the read length of 2 × 100 bp at the GeneCore SU, Sahlgrenska Academy Gothenburg University.

## 4 BIOINFORMATIC ANALYSES

### 4.1 Preprocessing

The reads were quality checked using Fastqc (0.11.2).^47^ The raw reads were quality filtered using trim galore (0.4.0).^48^

### 4.2 Human transcript analysis

The quality filtered reads were mapped towards the human reference genome GRCh38.90 using STAR (2.5.2b).^49^ The gene counts were calculated with featureCounts (1.6.4).^50^

The statistical analysis was performed with the R (4.0.1) package DESeq2(1.28.1).^51^, ^52^ The counts were normalized with size factors (ratio method).^53^ The data was transformed with variance stabilizing transformation.^53^ Outlier samples were removed during the statistical testing based on Cook's distance method.^54^ The *p*‐values were calculated with Wald statistics and were adjusted for multiple testing using the Benjamini–Hochberg procedure and are expressed as false discovery rate (FDR).^55^ The estimated log2 foldchanges were shrunken using the normal method.^51^

The correlation of the normalized counts of the detected significant genes to gestational age at birth was calculated using Spearman's rank correlation.^56^ The *p*‐values were adjusted using the Benjamini–Hochberg procedure, expressed as FDR.^55^

The ability of a specific transcript to discriminate between women who delivered preterm (<37 + 0) versus at term (39 + 0 to 40 + 6) is described as area under the receiver operating characteristic (ROC) curve (AUC), sensitivity and specificity. These were calculated using Prism software (version 9.2.0).

A gene set enrichment analysis was performed including the entire gene list ordered by log2 foldchange using ReactomePA.^57^ The exponent was set as 2, and the number of permutations were set to 100 000.

### 4.3 Microbiome–taxonomy analyses

The quality filtered reads were taxonomically classified using Kraken2 (2.0.8)^58^ using the RefSeq^59^ database as a reference, with a confidence score of 0.9. This threshold was selected to reduce the amount of noise. The confidence score is defined as the amount of matching kmers for a species divided by the total amount of kmers for the same species. The species abundance estimation was improved using Bracken (2.5)^60^ with a signal threshold of 10, meaning that species with less than 10 reads were ignored.

The statistical analysis was performed with the R (4.0.1)^61^ package DESeq2 (1.28.1).^51^ The data were filtered to only include taxa with a count sum of at least 1 000. All entries corresponding to viral species, phages and parasites were removed. The counts were normalized with size factors using poscounts method, a method implemented in phyloseq^62^ to handle data in which many samples have the value zero. Through this method, a modified geometric mean is measured taking the *n*th root of the product of the non‐zero counts. The data was processed with variance stabilizing transformation. Outlier samples were removed during the statistical testing based on Cook's distance method.^54^ The *p*‐values were calculated with Wald statistics and were adjusted for multiple testing using the Benjamini–Hochberg procedure, expressed as FDR.^55^ The estimated log2 foldchanges were shrunk using the normal method.^51^

The bacterial load was calculated for each sample by dividing the total sum of bacterial reads by the total sum of all classified reads (human and microbial).

### 4.4 Microbiome generation of vaginal community state types and diversity indices

All samples were clustered based on relative abundance of the detected species using complete linkage clustering as implemented in the R function hclust. Based on these clusters, community state types (CSTs) were defined by the species dominating each cluster, counted as relative abundance within each sample. CSTs were numbered according to Ravel et al.^18^ and Freitas et al.^24^ CST V was included in CST IVa for all analyses, as it only consisted of a single sample. Simpson diversity and species richness were calculated from the count data using R (4.0.1).^61^ Simpson index was calculated for each sample using the Vegan ‘diversity’ function.^63^ Normalized species richness was calculated by subsampling 10 00 000 reads from each library and calculating the number of unique species among these subsampled reads, to remove effects of varying library sizes. Differences (*p* < .05) in diversity between groups were tested by *t*‐tests on the richness and Simpson diversity indices. Heatmaps were generated using the ‘heatmap.2′ function, part of the gplots R package.

### 4.5 Microbiome‐functional pathways

To identify affected microbial pathways and ontologies, the microbial reads were extracted using the Kraken2 classification and analysed with HUMAnN (3.0.0)^64^ by using the search mode of uniref90^65^ and pathway MetaCyc.^66^ The tables were unstratified and normalized using the copies per million (cpm) method.^64^ The differences in abundance of the MetaCyc pathways were compared using the Wilcoxon rank‐sum test. The *p*‐values were adjusted using the Benjamini–Hochberg procedure, expressed as FDR.^55^

The normalized gene table was translated into Gene Ontology‐terms, using the regrouped table script within HUMAnN.^64^ The statistically significant differential Gene Ontology‐terms were identified using the Wilcoxon rank‐sum test. The *p*‐values were adjusted using the Benjamini–Hochberg procedure, expressed as FDR.^55^

### 4.6 Correlation between human and microbial gene expression

The significant human transcripts were correlated with the 20 most common bacterial transcripts in all subjects as well as in the preterm and the term group separately. The correlation analysis was performed using Spearman's rank correlation.^56^ The *p*‐values were adjusted using the Benjamini–Hochberg procedure, expressed as FDR.^55^

## 5 RESULTS

**FIGURE 1:**
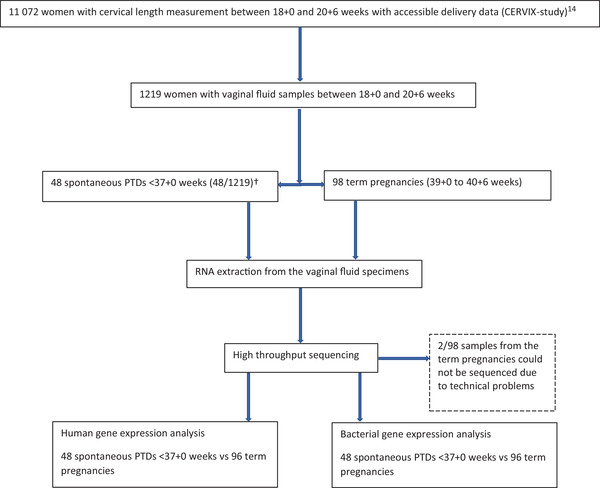
Flowchart showing the study design and group composition. PTD, preterm delivery. †Includes two spontaneous late miscarriages 18 + 0 to 21 + 6 after inclusion in the study

A flow chart showing the study design and study population is presented in Figure 1. Vaginal samples were collected from 1219 women. Of these, 46 women delivered spontaneously <37 + 0 gestational weeks and two had a late spontaneous miscarriage. Maternal characteristics were similar in the preterm and term group (Table 1). About 90% of the women were of white ethnicity and most had at least 12 years of education. The proportions of women with low socioeconomic status (education ≤9 years and/or unemployment and/or sick leave) and previous spontaneous PTD were higher in the preterm group compared to the term group. Characteristics regarding pregnancy, delivery and neonatal outcome are described in Table 2. The mean gestational age at delivery was 33 + 2 weeks in the preterm group and 40 + 1 weeks in the term group. No woman in either group had a redeemed prescription of progesterone or had a cerclage inserted after inclusion in the study.

**TABLE 1:** Baseline maternal characteristics

	**Preterm (sPTD <37 + 0** **weeks)**	**Term (delivery 39 + 0 to 40 + 6** **weeks)**	***p*‐value** **^‡^**
**Variable**	*n* = 48^†^	*n* = 96	
**Age at delivery (years)**	31.6 (4.7) *n* = 48	31.8 (4.3) *n* = 96	.84
**Ethnicity**			.60
**White**	45/48 (93.8%)	87/96 (90.6%)	
**Black**	0/48 (0.0%)	1/96 (1.0%)	
**Mixed White Black**	1/48 (2.1%)	0/96 (0.0%)	
**Middle East**	1/48 (2.1%)	2/96 (2.1%)	
**India**	0/48 (0.0%)	2/96 (2.1%)	
**Southeast Asian**	1/48 (2.1%)	2/96 (2.1%)	
**Other**	0/48 (0.0%)	2/96 (2.1%)	
**Maternal country of birth**			.16
**Sweden**	33/38 (86.8%)	78/88 (88.6%)	
**Other European**	4/38 (10.5%)	3/88 (3.4%)	
**Outside Europe**	1/38 (2.6%)	7/88 (8.0%)	
**Highest level of education**			.88
**9 years**	0/34 (0.0%)	1/83 (1.2%)	
**12 years**	12/34 (35.3%)	26/83 (31.3%)	
**>12 years**	22/34 (64.7%)	56/83 (67.5%)	
**Main occupation**			.11
**Employed**	27/38 (71.1%)	75/88 (85.2%)	
**Student**	5/38 (13.2%)	6/88 (6.8%)	
**Maternity leave**	1/38 (2.6%)	5/88 (5.7%)	
**Unemployed**	1/38 (2.6%)	1/88 (1.1%)	
**Sick leave**	3/38 (7.9%)	1/88 (1.1%)	
**Other**	1/38 (2.6%)	0/88 (0.0%)	
**Socioeconomic status**			.20
**Low socioeconomic status** **^§^**	4/38 (10.5%)	3/88 (3.4%)	
**Height at first antenatal visit (cm)**	166.1 (6.9) *n* = 38	167.0 (6.7) *n* = 80	.51
**BMI at first antenatal visit**	24.3 (4.1) *n* = 34	23.2 (3.2) *n* = 77	.14
**Smoking or using snuff at first antenatal visit**	3/45 (6.7%)	3/95 (3.2%)	.39
**Smoking at first antenatal visit**	2/36 (5.6%)	3/73 (4.1%)	1.00
**Alcohol screening performance (AUDIT)** **^¶^**			
**≥6 points (risk behaviour)**	4/36 (11.1%)	7/67 (10.4%)	.81
**IVF in current pregnancy**	1/46 (2.2%)	5/92 (5.4%)	.66
**Chronic hypertension at first antenatal visit**d	0/44 (0.0%)	0/88 (0.0%)	NA
**Diabetes type 1 or 2 at first antenatal visit**	1/44 (2.3%)	0/88 (0.0%)	.33
**Renal disease at first antenatal visit**	0/44 (0.0%)	0/88 (0.0%)	NA
**Conization prior to inclusion date**	3/48 (6.3%)	7/96 (7.3%)	1.00
**Parity**			
**Nulliparae**	27/48 (56.3%)	48/96 (50.0%)	.60
**Previous stillbirths ≥1**	0/48 (0.0%)	0/96 (0.0%)	NA
**Previous late miscarriage**	0/48 (0.0%)	0/96 (0.0%)	NA
**Previous spontaneous PTD < 37** **weeks ≥1**	6/48 (12.5%)	1/96 (1.0%)	.0057

For categorical variables *n* (%) is presented, for continuous variables Mean (SD) is presented.

Abbreviations: AUDIT, Alcohol Use Disorder Test; BMI, body mass index (kg/m^2^); IVF, in vitro fertilization; NA, not applicable; PTD, preterm delivery; sPTD, spontaneous preterm delivery.

^†^ Includes two late miscarriages 18 + 0 to 21 + 6 weeks after inclusion in the study.

^‡^ For comparison between groups Fisher´s Exact test (2 sided) was used for dichotomous variables, Mantel–Haenszel Chi Square test was used for ordered categorical variables, Chi Square test was used for non‐ordered categorical variables and *t*‐test was used for continuous variables.

^§^ Low socioeconomic status defined as education ≤9 years and/or unemployment and/or sick leave.

^¶^ Alcohol screening by AUDIT tool according to antenatal care routines.^67^

**TABLE 2:** Pregnancy, delivery, and neonatal outcome

	**Preterm (sPTD <37 + 0** **weeks)**	**Term (delivery 39 + 0 to 40 + 6** **weeks)**	***p*‐value** **^‡^**
**Variable**	*n* = 48^†^	*n* = 96	
**Redeemed prescription of vaginal progesterone after inclusion/18 + 0** **weeks until delivery**	0 (0.0%)	0 (0.0%)	NA
**Cerclage after inclusion**	0 (0.0%)	0 (0.0%)	NA
**Preeclampsia or gestational hypertension at delivery**	2 (4.2%)	2 (2.1%)	.60
**Chronic hypertension at delivery**	0 (0.0%)	0 (0.0%)	NA
**Diabetes type 1, 2 or gestational diabetes at delivery**	2 (4.2%)	0 (0.0%)	.11
**Mode of delivery**			.15
**Vaginal**	40 (87.0%)	93 (96.9%)	
**Caesarean delivery**	6 (13.0%)	3 (3.1%)	
**Gestational age at delivery (days)**	232.9 days (29.1) (33 + 2 weeks) *n* = 48	280.5 days (4.0) (40 + 1 weeks) *n* = 96	<.0001
**Late miscarriage before 22 + 0** **weeks**	2 (4.2%)	0 (0.0%)	.11
**Birth weight (g)**	2430 (699) *n* = 39	3561 (383) *n* = 94	<.0001
**Small for gestational age** ^§^ ^,1^	0 (0.0%)	2 (2.1%)	.55
**Apgar score at 5 min < 7**	5 (10.4%)	5 (5.3%)	.30
**Perinatal mortality** ^¶^	1 (2.1%)	0 (0.0%)	NA
**Stillbirth after 22 + 0** **weeks**	1 (2.1%)	0 (0.0%)	.33

For categorical variables *n* (%) is presented, for continuous variables Mean (SD) is presented.

Abbreviations: NA, not applicable; sPTD, spontaneous preterm delivery.

^†^ Includes two late miscarriages 18 + 0 to 21 + 6 weeks after inclusion in the study.

^‡^ For comparison between groups Fisher´s Exact test (2 sided) was used for dichotomous variables, Mantel–Haenszel Chi Square test was used for ordered categorical variables, Chi Square test was used for non‐ordered categorical variables and *t*‐test was used for continuous variables.

^§^ Defined as ≥2 SDs below the Swedish gestational age‐ and sex‐specific growth standard.^68^

^¶^ Includes stillbirth after 22 + 0 weeks and death within 7 days after birth.

### 5.1 Sequencing results

**FIGURE 2:**
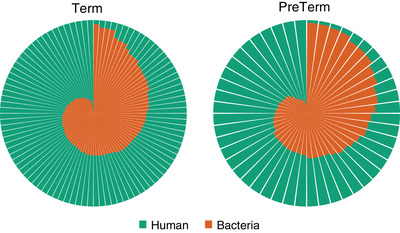
Stacked pie chart showing the proportion of human reads (annotated genes) and microbial reads (annotated microbes) in the preterm group (<37 + 0 weeks, *n* = 48) and the term group (*n* = 96). The proportion of human reads is shown in green, and the proportion of microbial reads in orange

The total dataset contained 8 487 635 189 quality filtered reads, and the mean amount of reads per sample was 58 941 911(range; 11 671 936 to 289 085 196). 4 907 632 710 reads were annotated as either human or microbial, with the proportion of human reads ranging from 3.1% to 98.9% (range; 74 537 to 9 882 152) (Figure 2).

There was no significant difference in bacterial load between the preterm (mean .45, 95% CI .39 to .52) and the term group (mean .39, 95% CI .36 to .43) (Figure S1). Nor was there an overall difference in species diversity (Simpson index: median .16, IQR .046 to .40 in the preterm group and median .16, IQR .06 to .38 in the term group) or richness (median 10, IQR 7 to 15.5 in the preterm group and median 11, IQR 8 to 15 in the term group) between the groups. (Figures S2 and S3).

### 5.2 Human genes and spontaneous PTD

**FIGURE 3:**
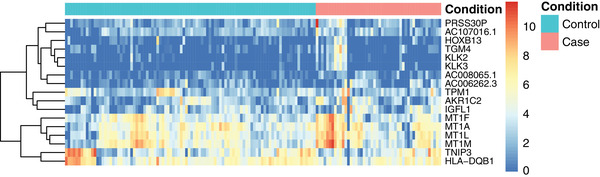
Heatmap showing the differentially expressed human genes (FDR *<* 0.05) in the preterm group (<37 + 0 weeks, *n* = 48) compared with the term group (*n* = 96). The analysis is based on log2 sizefactor normalized values from DESeq2^51^, ^52^, ^53^, ^54^ with an added pseudocount of +1. FC, fold change; FDR, false discovery rate (corrected *p*‐value for multiple comparison by Benjamini–Hochberg^55^); NS, non‐significant

To investigate whether there was an association between the expression of specific human genes and spontaneous PTD, we investigated the level of over‐and under expression (fold change) of each specific gene in the preterm and term group. A total of 17 human genes were significantly (FDR < 0.05) differentially expressed in the preterm group compared to in the term group. Of these 17 genes, 15 showed an overexpression in the preterm group compared to the term group (Table 3, Figure 3 and Figure S4). Human tissue kallikrein‐2(KLK2) was 10.6‐fold higher (FDR = 0.016); kallikrein‐3(KLK3) was 14.9‐fold higher (FDR = 0.019), the serine protease 30(PRSS30P) was 22‐fold higher (FDR = 0.003) and insulin growth factor‐like member 1(IGFL1) was 3.5‐fold higher (FDR = 0.016) in women who delivered preterm compared to those who delivered at term. Among the 17 differentially expressed genes there was a dominance of metallothioneins (MTs) (4/17), all showing an overexpression in the preterm group compared to the term group (FDR ranging between 0.009 and 0.048). A significant inverse correlation (FDR < 0.05) was seen between MT‐1L and gestational age at birth, as well as between KLK3 and gestational age at birth (Spearman's *ρ* : −0.27 and −0.24, respectively).

**TABLE 3:** Prevalence of significantly expressed human genes in women who delivered preterm and at term

			**Preterm (<37 + 0** **weeks, *n* = 48)**		**Term (*n* = 96)**		
**Human genes**	**Fold change** **^†^**	**FDR** **^‡^**	**Reads** **^§^**	**Prevalence (%)**	**Reads** **^§^**	**Prevalence (%)**	**Prevalence ratio**
AKR1C2	3.2	0.000	1723.27	37.50	1073.11	34.38	1.10
HOXB13	1.2	0.003	154.52	10.42	257.92	1.04	10.00
PRSS30P	22.2	0.003	3662.80	18.75	329.25	7.29	2.57
MT1L	2.8	0.009	5935.53	72.92	4226.49	75.00	−1.03
IGFL1	3.5	0.016	1504.35	37.50	870.67	29.17	1.29
KLK2	10.6	0.016	218.34	12.50	40.37	1.04	12.00
MT1M	3.1	0.016	9281.00	66.67	6022.29	72.92	−1.09
TGM4	15.5	0.016	613.33	10.42	78.09	1.04	10
TNIP3	−2.3	0.016	2645.89	66.67	12191.42	69.79	−1.05
AC107016.1	2.3	0.019	384.22	20.83	330.36	16.67	1.25
KLK3	14.9	0.019	193.07	14.58	25.05	0	14.58
TPM1	1.3	0.023	1616.58	31.25	2530.71	34.38	−1.10
MT1F	2.7	0.040	4560.34	60.42	3400.11	68.75	−1.14
AC008065.1	2.7	0.044	95.75	8.33	70.35	1.04	8
AC006262.3	3.8	0.048	383.08	18.75	200.80	7.29	2.57
HLA‐DQB1	−2.2	0.048	2417.27	83.33	10685.90	91.67	−1.10
MT1A	2.3	0.048	5920.52	83.33	5082.26	80.21	1.04

Abbreviation: FDR, false discovery rate.

^†^ Calculated by using the group sum of normalized values after group size correction. A gene is counted as present in a sample if the gene count is >9 (prevalence).

^‡^ False discovery rate represents the corrected *p‐*value for multiple comparisons between those who delivered preterm versus term (Benjamini–Hochberg^55^).

^§^ Normalized counts calculated with the ratio method^53^ within DESeq2. The sum of the normalized values is calculated for each gene within its group separately. Values are not corrected to group size.

We also tested to what extent the expressed human genes predicted spontaneous PTD. The transcript with the best AUC was AL031123.1 with an AUC of 0.70. At a threshold of 6.4 normalized read counts the sensitivity was 73% (95% CI, .63 to .81) and the specificity was 65% (95% CI, .50 to .77) (Figure S5).

### 5.3 Pathway analysis

To further examine the human gene expression data, we performed a gene set enrichment analysis, to evaluate potential differences in activated biological pathways in the preterm group compared to the term group. We identified 10 differentially activated biological pathways, and five of those are involved in the immune defence. An increased activity was seen in the metallothionein's bind metal pathway (FDR = 0.017) (Table 4).

**TABLE 4:** Gene set enrichment analysis showing the significant differences between women who delivered preterm and at term

**ID**	**Description of pathway**	**Set size**	**NES** **^†^**	***p*‐value**	**FDR** **^‡^**
R‐HSA‐8953854	Metabolism of RNA	649	−1.41	2.03e‐05	0.017
R‐HSA‐8868773	rRNA processing in the nucleus and cytosol	188	−1.53	2.11e‐05	0.017
R‐HSA‐6791226	Major pathway of rRNA processing in the nucleolus and cytosol	178	−1.53	3.18e‐05	0.017
R‐HSA‐72312	rRNA processing	197	−1.53	4.22e‐05	0.017
R‐HSA‐5661231	Metallothioneins bind metals	10	2.01	4.92e‐05	0.017
R‐HSA‐428543	Inactivation of CDC42 and RAC1	8	−1.64	6.42e‐05	0.02
R‐HSA‐1280215	Cytokine signalling in immune system	770	−1.33	0.0001	0.02
R‐HSA‐6809371	Formation of the cornified envelope	109	1.99	0.0001	0.03
R‐HSA‐913531	Interferon signalling	179	−1.49	0.0001	0.03
R‐HSA‐6798695	Neutrophil degranulation	464	−1.36	0.0002	0.05

Abbreviations: FDR, false discovery rate; ID, identification number.

^†^ NES, enrichment score normalized to mean enrichment of random samples based on the number of genes in the set.

^‡^ False discovery rate represents the corrected *p‐*value for multiple comparisons between those who delivered preterm < 37 + 0 weeks versus at term (Benjamini–Hochberg^55^).

### 5.4 Bacterial species and spontaneous PTD

To identify associations between individual taxa and spontaneous PTD, we investigated the abundance and prevalence of specific bacterial species in the preterm and term group. A total of 11 species showed significantly (FDR < 0.05) different gene expression in the preterm group compared to the term group. Ten of these eleven species had a low occurrence, together representing 0.8% of the total microbial reads in the dataset (Table 5). Out of the 11 species showing significantly different gene expression (FDR ranging between <0.001 and 0.002), eight had a higher prevalence in the term group. For example, *Bifidobacterium breve* transcripts were 2.5 times more common in the term group compared to the preterm group, and *Enterococcus faecalis* was 1.7 times more prevalent in the preterm group compared to the term group. *Lactobacillus crispatus* had higher expression levels in the preterm group (FDR = 0.001) but was less prevalent than in the term group (Table 5, Figure S6).

**TABLE 5:** Prevalence of bacteria significantly differentially expressed in the vaginal microbiome in women who delivered preterm and at term

			**Preterm (<37 + 0** **weeks, *n* = 48)**		**Term (*n* = 96)**		
**Species**	**Fold change** **^†^**	**FDR** **^‡^**	**Reads** **^§^**	**Prevalence (%)**	**Reads** **^§^**	**Prevalence (%)**	**Prevalence ratio**
*Bifidobacterium breve*	−1002.6	0.000	1029.61	6.25	2066584.47	15.63	−2.50
*Enterococcus faecalis*	−1862.8	0.000	1206.48	10.42	4498529.61	6.25	1.67
*Bacillus cereus*	−35.5	0.000	19314.35	6.25	1371036.08	10.42	−1.67
*Streptococcus agalactiae*	−402.3	0.000	59355.55	2.08	47752621.80	3.13	−1.50
*Lactobacillus fermentum*	−44149.3	0.000	0	0	88297.52	7.29	−7.29
*Lactobacillus plantarum*	−6522140.2	0.000	0	0	13044279.49	4.17	−4.17
*Campylobacter fetus*	−31523.1	0.000	0	0	63045.21	2.08	−2.08
*Parvimonas micra*	−1.1	0.000	1264.01	10.42	2902.75	10.42	1.00
*Xanthomonas vesicatoria*	−117579.1	0.000	0	0	235157.28	3.125	−3.13
*Lactobacillus crispatus*	4.8	0.001	967836444.5	93.75	404489545.50	96.88	−1.03
*Cutibacterium acnes*	−19.7	0.002	130.54	6.25	5177.77	16.67	−2.67

Abbreviation: FDR, false discovery rate.

^†^ Calculated by using the group sum of normalized values after group size correction. A microbe is counted as present in a sample if the microbe count is >9 (prevalence).

^‡^ False discovery rate represents the corrected *p‐*value for multiple comparisons between those who delivered preterm versus term (Benjamini–Hochberg^55^).

^§^ Normalized counts calculated with the poscounts method^62^ within DESeq2. The sum of the normalized values is calculated for each microbe within its group separately. Values are not corrected to group size.

**FIGURE 4:**
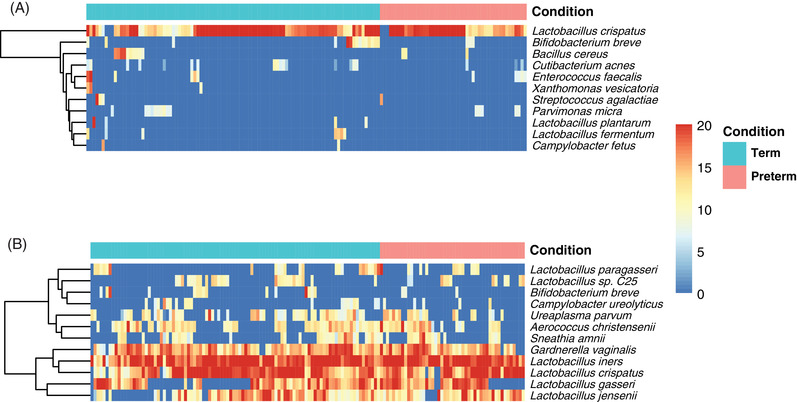
The expression of individual bacterial taxa. (A) Heatmap showing the differentially expressed bacteria (FDR *<* 0.05) in the preterm group (<37 + 0 weeks, *n* = 48) and the term group (*n* = 96). (B) Heatmap showing the ten most commonly expressed bacterial species in the preterm (<37 + 0 weeks, *n* = 48) and term group (*n* = 96), with a total sum of 12 species. Both heatmaps are based on log2 sizefactor normalized values from DESeq2 (poscounts method)^62^ with an added pseudocount of +1. FDR, false discovery rate (corrected *p*‐value for multiple comparison by Benjamini–Hochberg^55^)

The 10 most common bacterial species in the preterm and the term group are shown in Figure 4 and Table S1. The distribution of the most common bacteria was similar in the groups with a dominance of *Lactobacillus iners* and *L. crispatus*. However, *B. breve* was one of the 10 most common bacteria in the term group but not in the preterm group.

In order to evaluate the relationship between the human and the bacterial gene expression, we performed a Spearman's rank analysis. However, no significant correlations were found between the significantly differentially expressed human transcripts and the 20 most common bacterial transcripts in the preterm and the term group.

We also investigated whether any differences could be seen in the Gene Ontology‐analysis of the microbial genes in the preterm group compared to the term group, but no differences were found.

### 5.5 Vaginal community state types

**FIGURE 5:**
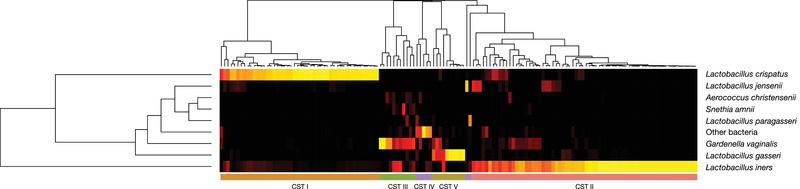
Heatmap showing the five identified vaginal community state types (CSTs), defined by their dominant species and counted as relative abundance within each sample. Note that CST IVa (purple) is divided across two different clusters as the CSTs were defined by their dominant species and CST IVa is the cluster with a diversity of dominant species (or without any dominant species in the sample). For all analyses the CST Iva also include the single sample classified as CST V

**FIGURE 6:**
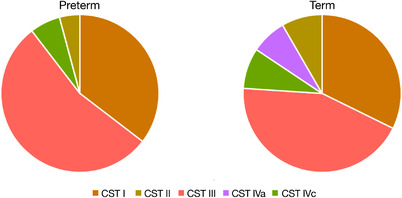
Stacked pie chart showing the distribution of the identified vaginal community state types (CSTs) in the preterm group (<37 + 0 weeks, *n* = 48) and the term group (*n* = 96). No significant difference was found apart from the absence of CST IV (mixture of different species) in the preterm group. For the preterm group CST I represented 36%, CST II 4%, CST III 54%, CST IVa 0% and CST IVc 6%, respectively. The corresponding numbers for the term group were 32% (CST I), 8% (CST II), 44% (CST III), 7% (CST IVa) and 9% (CST IVc)

We identified five major vaginal CSTs, defined by the dominance of either one species of *Lactobacillus*, *G. vaginalis* or a mixture of bacterial species. Three CSTs were dominated by either *L. crispatus* (CST I), *Lactobacillus gasseri* (CST II) or *L. iners* (CST III), one by *G. vaginalis* (CST IVc), and one by a variety of different species, for example, *Streptococcus agalactica*, *E. faecalis* and *B. breve* (CST IVa) (Figure 5). A single sample was classified as CST V (dominance by *Lactobacillus jensenii*). There was no difference in the overall CST distribution between the preterm and term group, apart from the absence of CST IVa (mixture of different species) in the preterm group (Figure 6). Most of the microbial profiles from the preterm group were assigned to the *Lactobacillus* dominated CSTs: CST I (36%), CST II (4%) and CST III (54%). The respective numbers for the term group were 32% (CST I), 8% (CST II) and 44% (CST III). Among those individuals with a dominance of *G. vaginalis* (CST IVc), assessment of alpha‐diversity (Simpson diversity index) revealed a significantly larger diversity (*p* < .05) in the preterm group compared to the term group. No differences in diversity were observed for the other CSTs.

## 6 DISCUSSION

We found 17 human genes to be differentially expressed in women who gave birth preterm compared to women who gave birth at term. Gene expression of KLK2 and KLK3 was higher in the preterm group, and there was also a general overexpression of MT1s in women who delivered preterm. In our mainly white population, there were no statistically significant differences in either bacterial load, diversity, or CST distribution between women who gave birth preterm versus at term. However, we found 11 individual bacterial species to be differentially expressed, most of them with a low occurrence. The frequent *L. crispatus* was shown to be more abundant in the preterm group compared to the term group.

To our knowledge, this is the first study to compare both the human and bacterial transcript profiles in vaginal fluid in women with a singleton pregnancy who delivered preterm versus at term, using high throughput sequencing. We have done this in a comparatively large patient cohort. Other strengths are the blinded prospective multicentre design of the study, strict validation of maternal characteristics and delivery outcomes, homogeneity regarding ethnicity and socioeconomic status of the study participants, and that no participant received progesterone or cerclage treatment.

A limitation is that we performed vaginal fluid sampling at only one time point. This means that we could not study possible longitudinal and interrelational changes between human and microbial transcriptional activity. Secondly, it could be seen as a limitation that we did not study the relationship between the total bacterial composition and the activity of the bacteria in the same vaginal specimens. Because the metatranscriptomic field is less explored than the metagenomic field, it would have been advantageous to study both. The lack of power (despite our relatively large study population) to perform subgroup analyses is a third limitation (e.g., separate analysis of early and late spontaneous PTD, or separate analysis of spontaneous onset of labour by contractions and preterm prelabour rupture of membranes). A general comment is that there are still large gaps in the annotation of microbial genes in comparison to human genes. This limits the ability to study functional pathways of the bacterial metatranscriptome and makes it difficult to interpret the results of such studies.^69^

It is widely accepted that intrauterine infection and inflammation are important causative factors of spontaneous PTD, which is often hypothesized to be secondary to pathogen ascension from the vagina.^7^, ^70^, ^71^, ^72^ Therefore, multiple studies have tried to characterize the relationship between the cervicovaginal microbiome and spontaneous PTD. However, the results diverge.^24^, ^25^, ^26^, ^27^, ^28^, ^29^, ^30^, ^31^, ^32^, ^33^, ^34^, ^35^ Both the microbiome itself and the relation between the microbiome and spontaneous PTD seem to differ depending on race and ethnicity.^18^, ^26^, ^27^ It has been proposed that *Lactobacillus* depletion is more common and species diversity higher in African American women than in white women,^25^, ^26^ but that a deficit of *Lactobacillus* and higher species diversity is a risk factor for spontaneous PTD primarily in white women and not in African American women.^26^, ^32^ Our results stand in contrast to those of other studies including mainly white women^24^, ^25^ in that we saw no difference in bacterial diversity or richness between women who delivered preterm compared to women who delivered at term. Nor did *L. crispatus* seem to be a protective factor against spontaneous PTD in our population, despite *L. crispatus* having been proposed to be protective in three other studies with a mainly white population.^28^, ^29^, ^34^ However, comparison is difficult, because in most other studies a DNA‐based assessment of bacterial community composition was used,^24^, ^25^, ^28^, ^29^, ^34^ while we used a metatranscriptomic approach. Studies using DNA‐based analysis provide information on the taxonomic composition of the bacterial communities but not on whether the bacteria are active members of the microbiome. Sequencing of the metatranscriptome describes the activity of microbial communities.^36^ France et al. demonstrated that the presence of species discovered by a metagenomic approach is not always indicative of its transcriptional activity, and that impending changes in the vaginal bacterial community can be predicted with a metatranscriptomic approach.^73^ This implies that taxa observed at higher relative abundance in a DNA‐based assessment could be under‐represented in the metatranscriptome. Fettweis et al. demonstrated overrepresentation of *L. crispatus* in the term compared with the preterm group in their metatranscriptomic analysis. This suggests that it is not a difference in methodological approach that explains the discrepancy for this strain. The metatranscriptomic results obtained by Fettweis et al. agree with ours in that it was mainly microbes of low occurrence that differed between the preterm and term group, and both studies found that the expression of *B. breve* was higher in the term than in the preterm group. Otherwise, there was poor agreement between the two studies for microbes of low occurrence. This can at least partly be explained by differences in study design: the women in the study of Fettweis et al. were sampled at 25‐26 weeks of gestation (our cohort was sampled at 18‐20 weeks), were mainly black (our women were predominantly white), and a significant proportion of women received progesterone or cerclage treatment in the study by Fettweis et al. (our cohort was not treated).^31^

Even though we did not find any significant correlation between transcriptionally active bacterial species and human genes, several interesting human genes were found to be transcriptionally enhanced in the preterm group compared to the term group. Of these, the most significant are KLK2, KLK3, several isoforms of MT1s, PRSS30P and IGFL1. KLK2 and KLK3 are part of the tissue kallikrein‐related peptidase family consisting of 15 proteolytic enzymes, which are highly influenced by pH.^74^ The marked increase of PRSS30P could be important, since PRSS30P belongs to the serine protease family, which has been shown to exert KLK enzymatic activity in inflammatory processes.^75^ Insulin‐like growth factor‐1 (IGF1) proteins are known to be involved in major biological processes such as growth and reproduction^76^ and have been linked to preterm delivery.^77^ IGF1L (with an elevated transcription in the preterm group) has shown a structural similarity with IGF1 protein and is thought to have growth modulating effects similar to IGF1.^78^ KLK2 and KLK3 have been proposed to degrade several IGF‐binding proteins, thereby controlling the level of biologically active IGF1,^79^ but it is still unknown to what extent KLKs modulate IGF1L. It has been postulated that KLKs could be associated with important events linked to PTD such as: (1) activation of antimicrobial peptides, (2) degradation of the cervical mucus plug by cleaving mucin proteins, and (3) breakdown of the foetal membranes by degradation of extracellular matrix components such as collagens, fibronectin and lamines.^80^, ^81^ Muytjens et al. summarize that the KLK activity in vaginal fluid is expected to be low in healthy women of reproductive age because of the physiologically low vaginal pH but that a disturbance in this balance, either by changes in the vaginal microbiome, decrease in oestrogen levels or mixing of vaginal fluid with fluids of increased alkalinity (e.g., amniotic fluid) could increase KLK activity.^80^ Members of the KLK family are associated with both inflammation and cancer. They are already in use, or have been proposed to be used, as potential biomarkers and therapeutic targets in several types of cancer.^82^ We found a weak but significant correlation between KLK2 and gestational age at birth. This raises the question whether members of the KLK‐family proteins could function as biomarkers for prediction of spontaneous PTD, perhaps in conjunction with second trimester cervical length measurement. Further studies are needed to characterize the KLK enzymatic activity in vaginal specimens in relation to the occurrence of spontaneous PTD.

Another interesting finding in our study is the overexpression of four different isoforms of MT1s and the increased activation of the metallothioneins binding metal pathway in those who delivered preterm compared to those who delivered at term. MTs play an important role in regulating oxidative stress, inflammation and hormone signalling.^83^, ^84^ There is limited information on the role of MTs in spontaneous PTD. A small study by Lappas et al. demonstrated that MT1‐4 was upregulated in intrauterine tissues in women with PTD complicated by bacterial infection.^85^ It has also been shown that MT1A is expressed in the foetal membranes in women delivering preterm^86^ and MT1A was higher in membranes close to the rupture site compared with more distant sites in women with spontaneous rupture of the membranes at term.^87^ The expression of MT1L was higher in the membranes in women delivering preterm in pregnancies complicated by chorioamnionitis.^88^ It is therefore interesting that we detected elevated expression of MT1A, MT1L and MT1M in vaginal samples at 18 + 0 to 20 + 6 weeks, that is, several weeks before spontaneous PTD. Another study has reported an increased expression of MT1A and MT2A in the myometrium of women with arrest of cervix dilatation during labour. The authors attributed this to an increase in reactive oxygen species due to oxidative stress.^90^ Indeed, oxidative stress is an important component also in the inflammatory reaction leading to preterm birth. This is supported by several studies demonstrating that superoxide dismutase 2 is upregulated in gestational tissues in preterm labour.^89^ Hypothetically, increased expression of MTs could be a marker of reactive oxygen species activation associated with PTD induced by infection or inflammation.

Some of the transcripts predicted preterm delivery. Expression of AL031123.1 discriminated between women who subsequently gave birth preterm versus at term with an AUC of 0.70. An AUC of 0.70 indicates only a moderate discriminative ability, and expression of AL031123.1 is unlikely to be clinically useful. AL031123.1 is a long non‐coding sequence with unknown function expressed in renal carcinoma cells.^91^

## 7 CONCLUSION

We present novel information on the transcriptionally active human and microbial genes in vaginal fluid, assessed by high throughput sequencing, in women with a singleton pregnancy who delivered <37 + 0 weeks or at term. Our findings could contribute to the understanding of the process leading to spontaneous PTD. Whether the KLK and MT genes or their respective translated proteins are biomarker candidates for spontaneous PTD needs to be explored. The lack of major differences in the active microbiome between women who subsequently give birth preterm versus at term, suggests that the metatranscriptome has a limited ability to serve as a diagnostic tool for identification of those at high risk for preterm delivery.

## CONFLICT OF INTEREST

The authors declare no conflict of interest.

## Supporting information

Supporting Information

Supporting Information

Supporting Information

Supporting Information

Supporting Information

Supporting Information

Supporting Information

Supporting Information

Supporting Information

Supporting Information

## Data Availability

The datasets generated for the metatranscriptomic data in the current study will be available in the European Nucleotide Archive (ENA) at EMBL‐EBI under accession number PRJEB50547, [https://www.ebi.ac.uk/ena/browser/view/PRJEB50547]. Data sharing of the human transcriptomic data is not possible because of the risk of compromising the privacy of the individual and because of Swedish data integrity laws. A record of the bioinformatic analysis is included as Additional file 8 and 9, and was created using the knitr package in R. Metadata needed for analysis have been included as Additional files 10, 11, 12, 13 and 14, respectively.
