# Supplementary material for: Microbial and human transcriptome in vaginal fluid at midgestation: Association with spontaneous preterm delivery
Source: Clin Transl Med. 2022 Sep 14;12(9):e1023. doi: 10.1002/ctm2.1023 (PMC9473488; doi:10.1002/ctm2.1023)
Supplement: Supplementary file 1 — Supporting Information [file CTM2-12-e1023-s009.pdf]

Supplementary information for

Microbial and human transcriptome in vaginal fluid at midgestation: association with spontaneous preterm delivery

Tove Wikström[1, 2], Sanna Abrahamsson[3], Johan Bengtsson-Palme[4, 5, 6], C. Joakim Ek[7], Pihla Kuusela[8], Elham Rekabdar[3], Peter Lindgren[9, 10], Ulla-Britt Wennerholm[1, 2], Bo Jacobsson[1, 2], Lil Valentin[11, 12], Henrik Hagberg[1, 2]

[1]Centre of Perinatal Medicine and Health, Department of Obstetrics and Gynecology, Institute of Clinical Sciences, Sahlgrenska Academy, University of Gothenburg, Gothenburg, Sweden

[2]Region Västra Götaland, Sahlgrenska University Hospital, Department of Obstetrics, Gothenburg, Sweden

[3]Bioinformatics Core Facility, Sahlgrenska Academy, University of Gothenburg, Sweden

[4]Department of Infectious Diseases, Institute of Biomedicine, Sahlgrenska Academy, University of Gothenburg, Sweden

[5]Centre for Antibiotic Resistance Research (CARe) at University of Gothenburg, Sweden

[6]Division of Systems and Synthetic Biology, Department of Biology and Biological Engineering, Chalmers University of Technology, SE-412 96, Gothenburg, Sweden

[7]Institute of Neuroscience and Physiology, Department of Physiology Sahlgrenska Academy, University of Gothenburg, Sweden

[8]Södra Älvsborg Hospital, Borås, Sweden

[9]Department of Clinical Science, Intervention and Technology, Karolinska Institutet, Stockholm, Sweden

[10]Centre for Fetal Medicine, Karolinska University Hospital, Stockholm, Sweden

[11]Department of Obstetrics and Gynecology, Skåne University Hospital, Malmö, Sweden

[12]Department of Clinical Sciences Malmö, Lund University, Sweden

Corresponding Author

Tove Wikström, specialist in Obstetrics and Gynecology

ORCID ID: 0000-0002-4208-8667

Department of Obstetrics and Gynecology

Sahlgrenska University Hospital

416 50 Gothenburg

Sweden

Telephone: +46 707813447

Email: tove.wikstrom@vgregion.se

Funding

The study was funded by The Swedish Research Council (Dnr 2014-06998; Dnr 2019-01320), Forskning och Utbildning (FoU) Södra Älvsborg, by the Swedish state under the agreement between the Swedish government and the county councils, the ALF-agreement (ALFGBG-136431, ALFGBG-426411, ALFGBG-718591), and The Swedish National Patient Insurance Company (LÖF). The funders had no role in study design; in the collection, analysis, and interpretation of data; in the writing of the report; and in the decision to submit the work for publication. The researchers were independent of the funders.

Contents:

Supplementary Table 1. Prevalence of the 20 most common bacteria in all women and in women who delivered preterm or at term — Page 4-5

Supplementary Figure 1. Bacterial load — Page 6

Supplementary Figure 2. Simpson Diversity Index — Page 7

Supplementary Figure 3. Normalized species richness — Page 8

Supplementary Figure 4. Volcano plot illustrating the differentially expressed human genes — Page 9

Supplementary Figure 5. Receiver operating characteristic curve — Page 10

Supplementary Figure 6. Volcano plot illustrating the significantly differentially expressed bacterial species — Page 11

Additional file 6. General description of the bioinformatic analyses — See separate file

Additional file 7. Record of the bioinformatic analyses for diversity and CST performed in R — See separate file

Additional file 8. Record of the bioinformatic analyses performed in R — See separate file

Additional file 9. Record of the bioinformatic analyses performed in R (diversity and CST) — See separate file

Additional file 10. Metadata associated with all samples used in this study (preterm or term) — See separate file

Additional file 11. Metadata associated with all samples used in this study (gestational days) — See separate file

Additional file 12. Summary of all raw human counts See separate file

Additional file 13. Summary of all raw microbial counts See separate file

Additional file 14. Metadata for calculation of bacterial load See separate file

Supplementary Table 1. Prevalence of the 20 most common bacteria in all women and in women who delivered preterm or at term

Species	Fold Change †	FDR ‡	Preterm (<37+0 weeks, *n*=48)		Term (*n*=96)		Prevalence ratio	All	Preterm	Term
			Reads§	Prevalence (%)	Reads§	Prevalence (%)				
Bifidobacterium breve	-1002.6	0.000	1029.61	6.25	2066584.47	15.63	-2.5	x		x
Enterococcus faecalis	-1862.8	0.000	1206.48	10.42	4498529.60	6.25	1.67	x		x
Streptococcus agalactiae	-402.3	0.000	59355.54	2.08	47752621.80	3.13	-1.50	x		x
Lactobacillus fermentum	-44149.3	0.000	0	0	88297.52	7.29	-7.29	x		x
Campylobacter fetus	-31523.1	0.000	0	0	63045.21	2.08	-2.08	x		x
Lactobacillus crispatus	4.8	0.001	967836444.5	93.75	404489545.50	96.88	-1.03	x	x	x
Gardnerella vaginalis	-15.1	0.080	19038533.75	91.67	573726807.40	95.83	-1.05	x	x	x
Lactobacillus jensenii	2.3	0.405	97641385.34	91.67	86318147.46	78.13	1.17	x	x	x
Lactobacillus reuteri	-36.4	0.461	1027.54	6.25	74841.25	8.33	-1.33	x		x
Campylobacter ureolyticus	-9.5	0.522	6969.22	10.42	132820.42	19.79	-1.90	x		x
Peptostreptococcaceae bacterium oral taxon 929	-3.2	0.686	6636.96	14.58	43121.91	23.96	-1.64	x		x
Lactobacillus paragasseri	8.4	0.868	3750134.93	25	892876.01	23.96	1.04	x	x	x
Sneathia amnii	3.2	0.912	1412339.10	37.50	891292.04	38.54	-1.03	x	x	x
Lactobacillus amylovorus	1.0	0.928	62945.37	22.92	124130.03	15.63	1.47		x	
Lactobacillus gasseri	-1.1	0.928	25134633.04	70.83	57342200.21	67.71	1.05	x	x	x
Lactobacillus iners	1.1	0.928	1523768276	100	2684012244	100	1	x	x	x
Lactobacillus sp. C25	1.2	0.928	125843.31	25	210196.85	28.13	-1.13	x	x	x

Species	Fold Change †	FDR ‡	Preterm (<37+0 weeks, *n*=48)		Term (*n*=96)		Prevalence ratio	All	Preterm	Term
			Reads§	Prevalence (%)	Reads§	Prevalence (%)				
Lactobacillus sp. JM1	-2.9	0.928	29725.10	8.33	170021.07	6.25	1.33	x	x	x
Lactobacillus vaginalis	1.0	0.928	88848.83	35.42	172896.21	28.13	1.26		x	
Mageeibacillus indolicus	-2.2	0.928	22682.35	33.33	101816.62	27.08	1.23		x	
Megasphaera stantonii	2.0	0.928	55915.12	29.17	55527.42	29.17	1		x	
Mycoplasma hominis	-4.8	0.928	53321.34	20.83	514157.26	14.58	1.43	x	x	x
Prevotella intermedia	-2.0	0.928	8657.84	18.75	34437.65	13.54	1.38		x	
Staphylococcus epidermidis	3.1	0.928	16019.08	12.50	10492.91	8.33	1.50		x	
Streptococcus salivarius	1.9	0.928	7795.39	6.25	8323.32	2.08	3		x	
Ureaplasma parvum	-1.9	0.928	345010.33	41.67	1295997.52	56.25	-1.35	x	x	x
Aerococcus christensenii	3.6	0.962	4121462.78	54.17	2272254.68	62.50	-1.15	x	x	x
Streptococcus mitis	5385.9	NA	2691.96	4.17	0	0	4.17		x	

FDR, false discovery rate
† Calculated by using the group sum of normalized values after group size correction. A microbe is counted as present in a sample if the microbe count is >9 (prevalence)
‡ False discovery rate represents the corrected *p*-value for multiple comparisons between those who delivered preterm vs term (Benjamini Hochberg[55])
§ Normalized counts calculated with the poscounts method[62] within DESeq2. The sum of the normalized values is calculated for each microbe within its group separately. Values are not corrected to group size.
X denotes the 20 most common bacteria in the respective group (i.e., all women, the preterm group, and the term group)

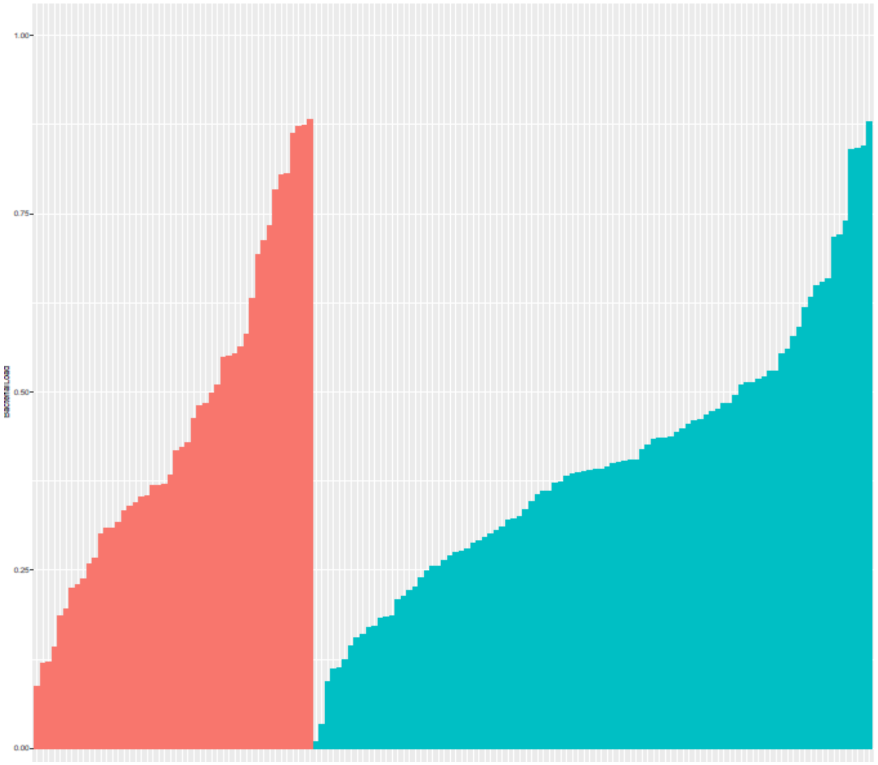

Supplementary Figure 1. Bacterial load in the preterm group (<37+0 weeks, *n*=48) and in the term group (*n*=96). The pink bars indicate the bacterial load in the preterm group (mean 0.45, 95% CI 0.39 to 0.52), and the blue bars indicate the bacterial load in the term group (mean 0.39, 95% CI 0.36 to 0.43). The bacterial load was calculated for each sample by dividing the total sum of bacterial reads by the total sum of all classified reads (human and microbial).

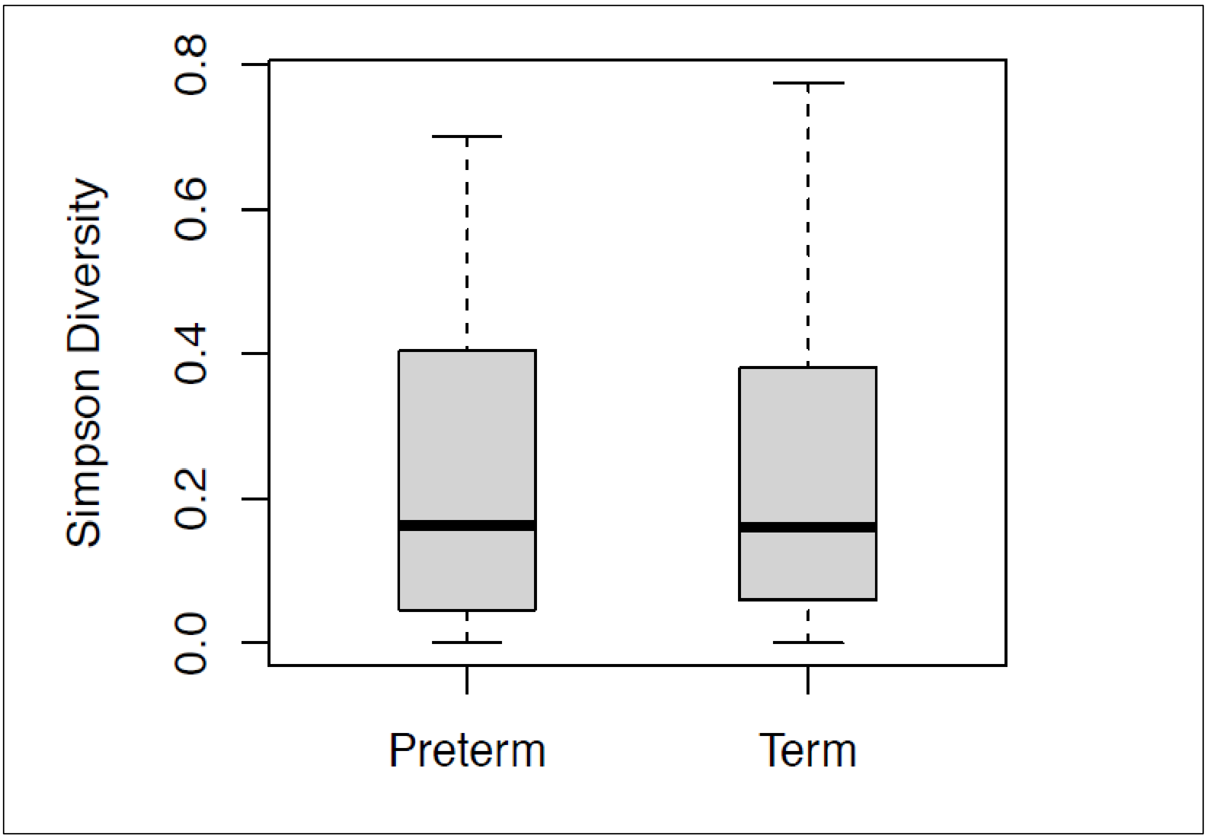

Supplementary Figure 2. Simpson Diversity Index in the preterm (<37+0 weeks, *n*=48) and term group (*n*=96). The figure displays the median (line inside the box), the interquartile range (delineated by the box) and the minimum and maximum values (whiskers).

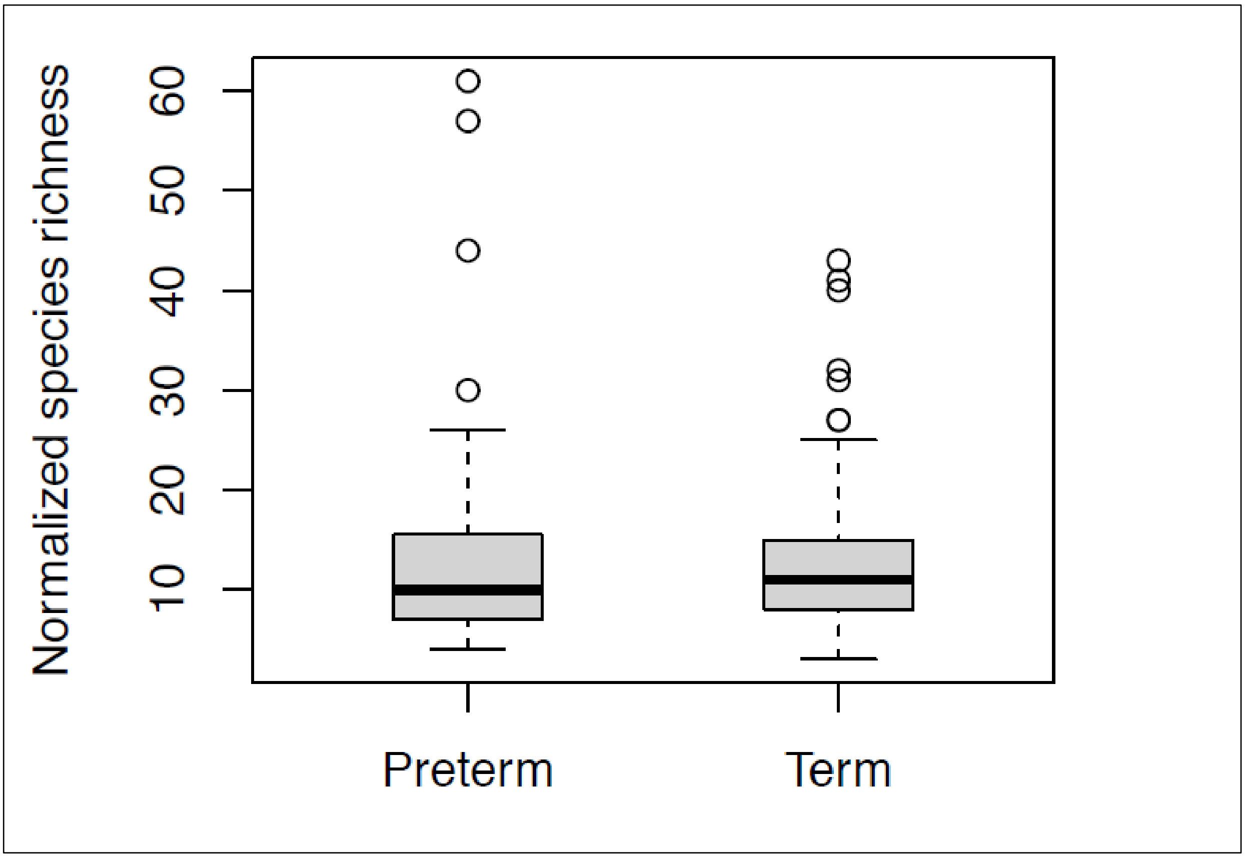

Supplementary Figure 3. Normalized species richness in the preterm (<37+0 weeks, *n*=48) and term group (*n*=96). The figure displays the median (line inside the box), the interquartile range (delineated by the box), and the values within 1.5 times the interquartile range (whiskers). Values outside the whiskers are outside of the 1.5 times interquartile range.

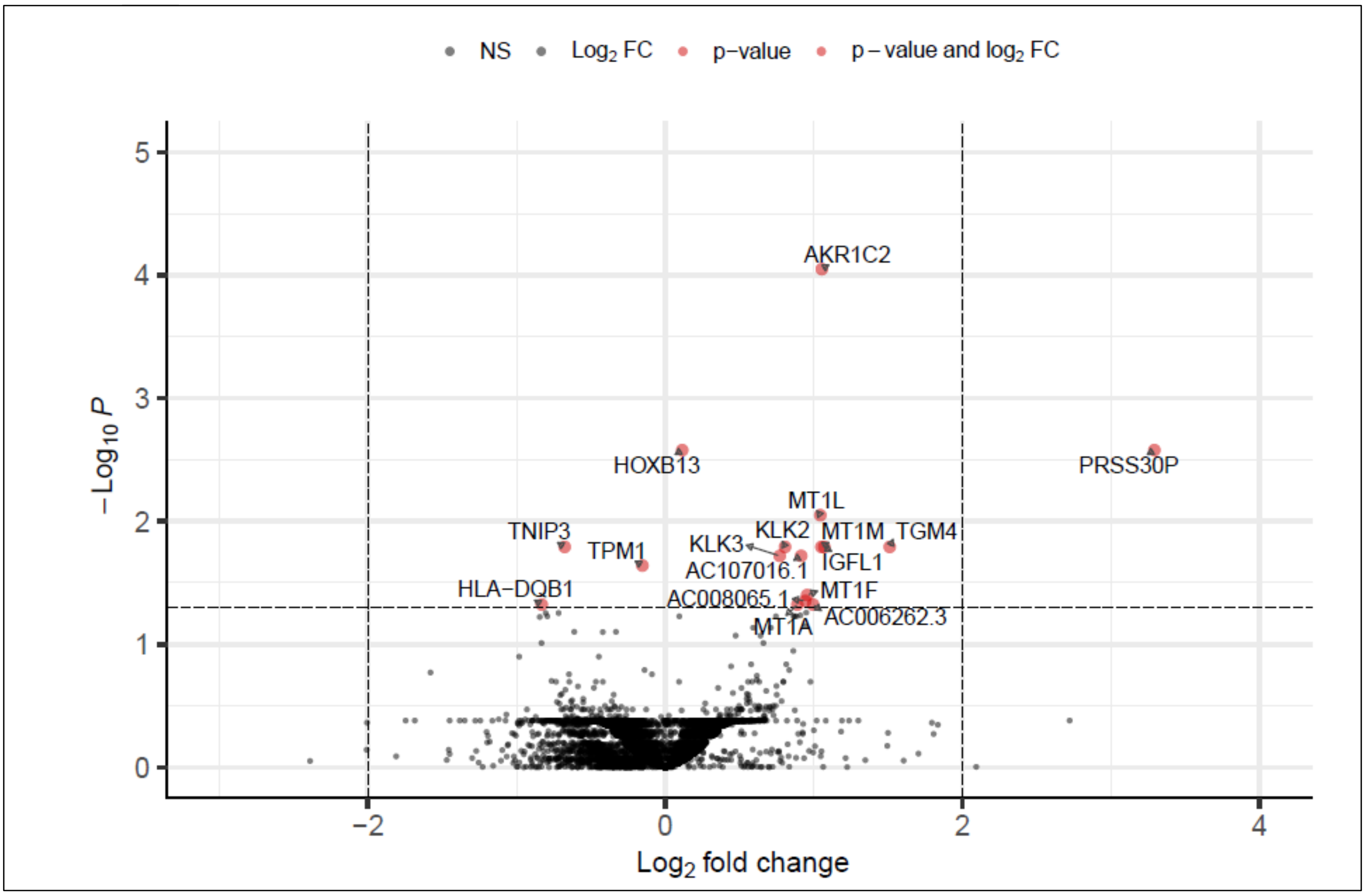

Supplementary Figure 4. Volcano plot illustrating the differentially expressed human genes which were significantly regulated (*FDR <0.05*) in the preterm group (<37+0 weeks, *n*=48) compared with the term group (*n*=96). The statistical significance is shown on the y-axis and the magnitude of change (fold change) is shown on the x-axis.

NS, non-significant; FC, fold change; FDR, false discovery rate (corrected *P*-value for multiple comparison by Benjamini Hochberg[55])

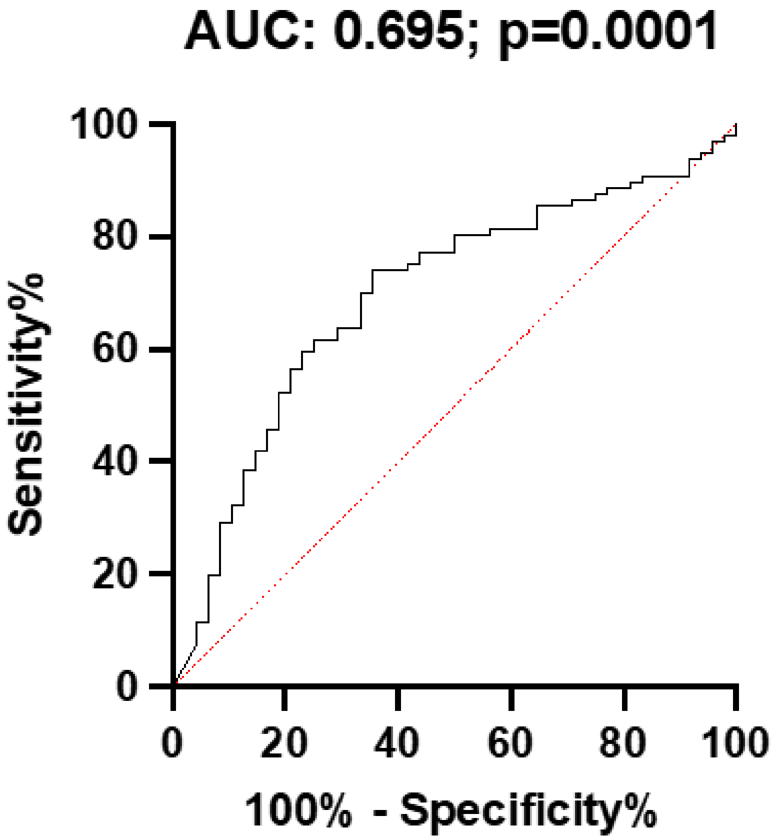

Supplementary Figure 5. Receiver operating characteristic curve illustrating the ability to discriminate between women who delivered preterm (<37+0 weeks) and at term for the transcript (AL031123.1) which had the largest area under the receiver operating characteristic curve (AUC) of all the expressed human genes.

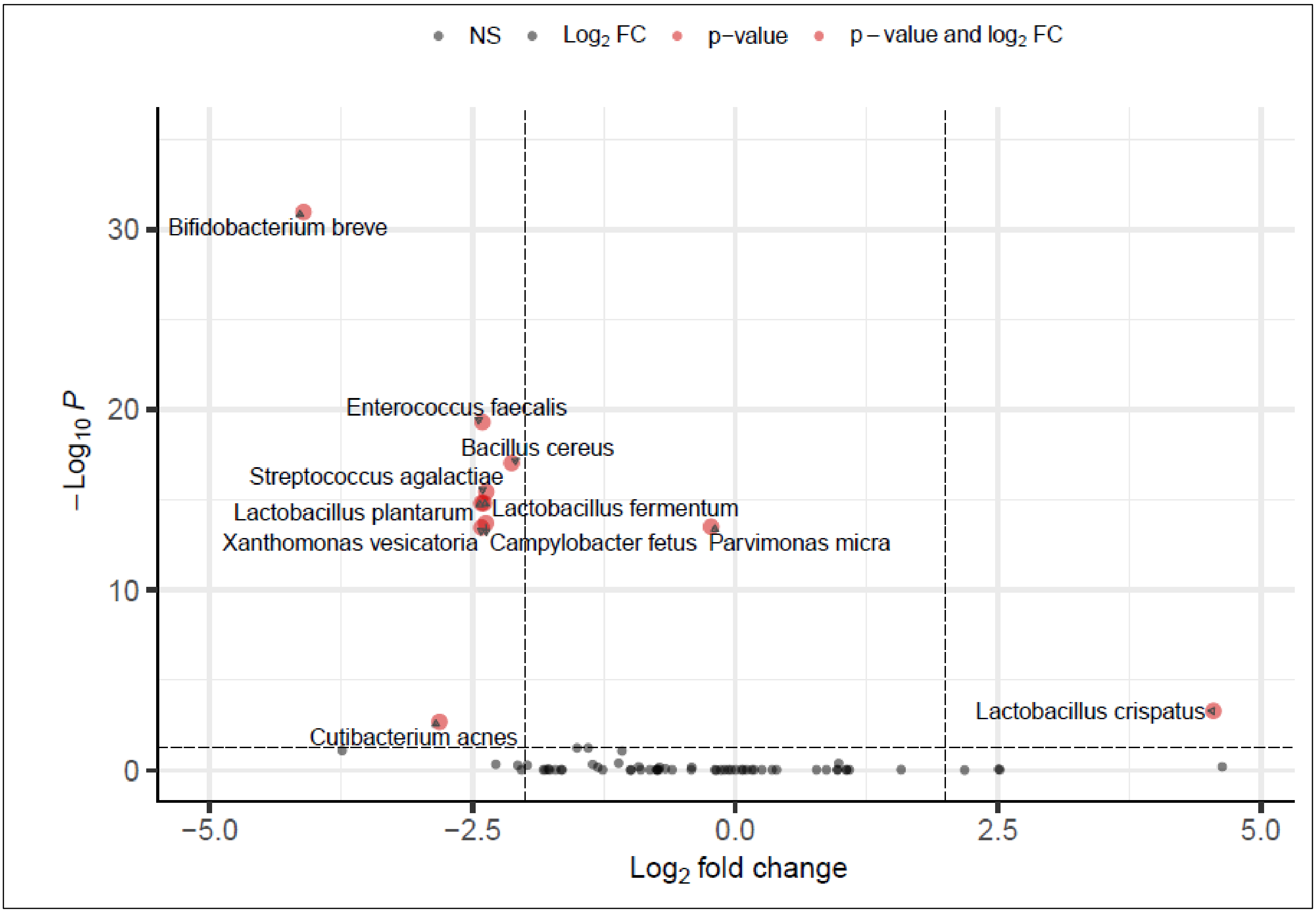

Supplementary Figure 6. Volcano plot illustrating the significantly (*FDR <0.05*) differentially expressed bacterial species in the preterm group (<37+0 weeks) compared with the term group. The statistical significance is shown on the y-axis and the magnitude of change (fold change) is shown on the x-axis.

NS, non-significant; FC, fold change; FDR, false discovery rate (corrected *P*-value for multiple comparison by Benjamini Hochberg[55])
