# Supplementary material for: Microbial and human transcriptome in vaginal fluid at midgestation: Association with spontaneous preterm delivery
Source: Clin Transl Med. 2022 Sep 14;12(9):e1023. doi: 10.1002/ctm2.1023 (PMC9473488; doi:10.1002/ctm2.1023)
Supplement: Supplementary file 2 — Supporting Information [file CTM2-12-e1023-s006.pdf]

Vaginal microbial and human transcriptome at midgestation: association with spontaneous preterm delivery

Vaginal microbial and human transcriptome at midgestation: association with spontaneous preterm delivery

- Background
- Human Transcript analysis
  - Plots
  - Spearman Correlation
  - GSEA
- Microbiome Taxonomy analysis
  - Plots
  - Microbiome reads vs human gene reads
  - Most common species plot
  - Bacterial Load plot (supplementary)

Background

Vaginal swaps looking into the expression of human transcripts and the microbial content.

Human Transcript analysis

Loading the required libraries

```
library(DESeq2)
library(magrittr)
library(pheatmap)
library(EnhancedVolcano)
library(AnnotationDbi)
library(org.Hs.eg.db)
library(reshape)
library(dplyr)
library("BiocParallel")
```

```
library(RColorBrewer)
register(MulticoreParam(4))
```

Load the count data and the Metadata and its metadata that defines the groups

```
Counts <- read.table("datafiles/RawCounts_Human_Renamed.txt", header = T, sep = "\t",
    row.names = 1)

metadata <- read.csv(file = "datafiles/Metadata_Renamed.txt", header = TRUE, sep = "\t",
    row.names = 1)
metadata <- metadata[colnames(Counts), , drop = FALSE]
```

Read to a DESeq2 object

```
dds <- DESeqDataSetFromMatrix(countData = Counts, colData = metadata, design = ~Condition)
```

Filter the object removing genes with only 1 count or less.

```
dds <- dds[rowSums(counts(dds)) > 1, ]
```

Running the DESeq2 analysis Comparing the Preterm vs Term

```
dds <- DESeq(dds, betaPrior = FALSE, , parallel = TRUE, BPPARAM = MulticoreParam(4))
```

Write normalized counts

```
normCounts <- counts(dds, normalized = TRUE)

write.table(normCounts, file = "outputs/NormalizedCounts_Human.txt", col.names = NA,
    row.names = TRUE, sep = "\t", quote = FALSE, na = "NA")
```

Calculate the vst (variance stabilization transformation)

```
vst <- vst(dds)
```

The results from the Case vs control comparison is extracted while the foldchanges are shrunken using the normal method. The genes are sorted by padjusted values.

```
lfshrink <- lfcShrink(dds, contrast = c("Condition", "Preterm", "Term"), type = "normal",
    parallel = TRUE, BPPARAM = MulticoreParam(4))
lfshrink <- lfshrink[order(lfshrink$padj), ]
```

Add the genename to the result matrix for a more detailed description

```
lfshrink$description <- mapIds(org.Hs.eg.db, keys = row.names(lfshrink), column = "GENENAME",
    keytype = "SYMBOL")
```

Write the result to a file

```
write.table(lfshrink, file = "outputs/Human_Preterm_vs_Term.txt", col.names = NA,
    row.names = TRUE, sep = "\t", quote = FALSE, na = "NA")
```

Plots

Making the volcano plot with labels of the significant genes

```
genesOfInterest <- rownames(lfshrink[which(lfshrink$padj < 0.05), ])

plot(EnhancedVolcano(lfshrink, lab = rownames(lfshrink), x = "log2FoldChange", y = "padj",
    title = "Case vs Control", pCutoff = 0.05, FCcutoff = 2, selectLab = genesOfInterest,
    legendIconSize = 2, drawConnectors = TRUE, widthConnectors = 0.5, colConnectors = "grey30",
    labSize = 5, ylim = c(0, 5), xlim = c(-3, 4), pointSize = c(ifelse(lfshrink$padj <
        0.05, 3, 1)), ))
```

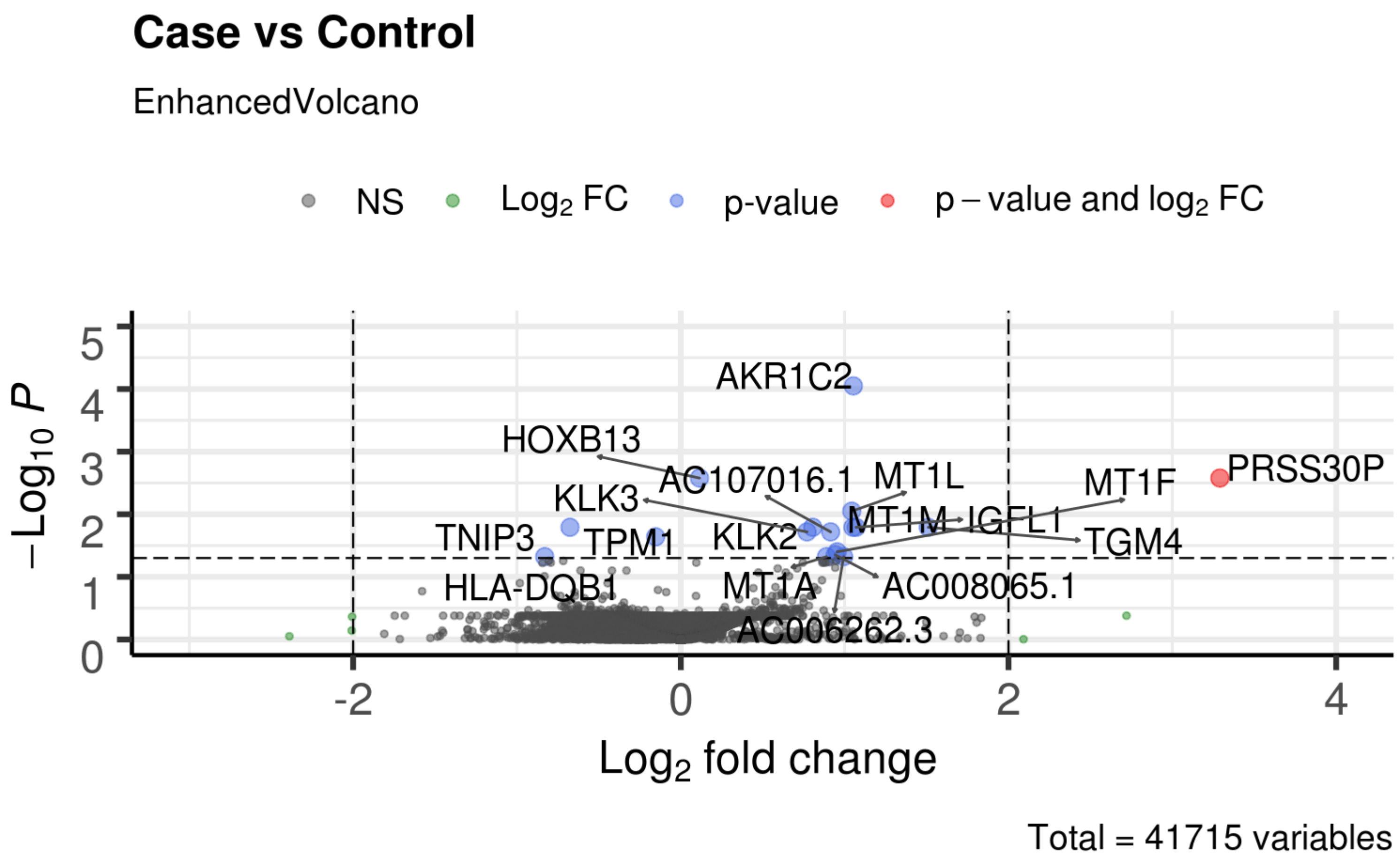

Creating the heatmap of the significant genes

```
library(Pigengene)

normCounts <- counts(dds, normalized = TRUE)
```

```r
signenesNorm <- normCounts[genesOfInterest, rownames(metadata[order(metadata$Condition,
    decreasing = T), , drop = FALSE])]
tsignenesNorm <- t(signenesNorm)
metadata$Condition <- factor(metadata$Condition, levels = c("Term", "Preterm"))
pheatmap.type(log2(tsignenesNorm + 1), annRow = metadata, doTranspose = TRUE, show_colnames = FALSE,
    conditions = c("Term", "Preterm"), cellheight = 7, fontsize_row = 7)
```

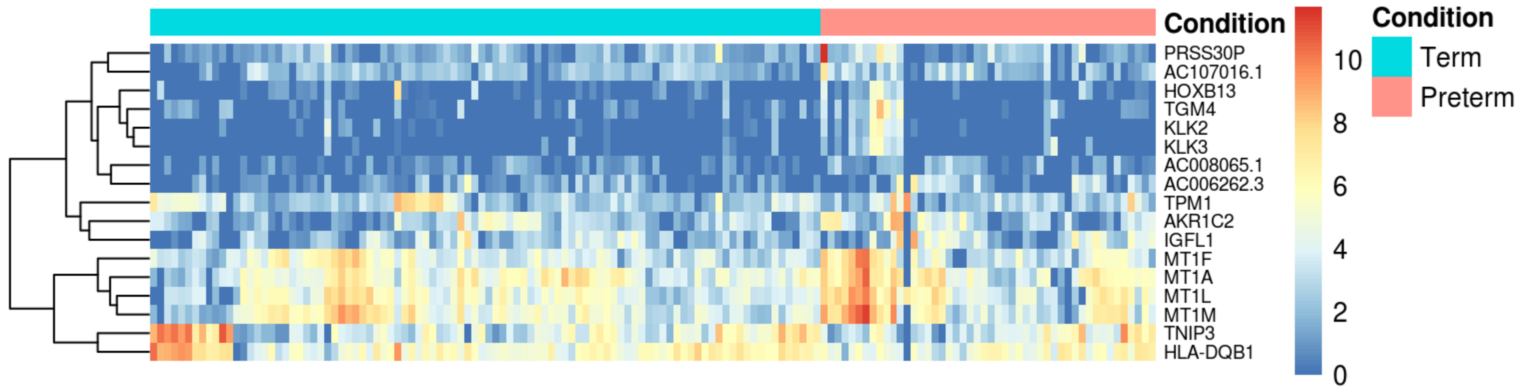

Spearman Correlation

Correlation between the significant genes and the gestational age

Read the file

```r
Ages <- read.csv("datafiles/GestitionAge_ALL_Renamed.txt", sep = "\t")
```

Make sure the order of the normalized genes are the same as the gestational ages

```r
Ages <- Ages[match(colnames(signenesNorm), Ages$X), ]
Ages <- Ages$Age_days
```

Run Spearman Correlation Comparing the normalized counts of the significant genes vs gestational age

```r
SpearmanCorrelation = c()
HUM = c()
Pvalues = c()
for (i in rownames(signenesNorm)) {
    Scor <- cor(as.numeric(Ages), as.numeric(signenesNorm[i, ]), method = "spearman")
    Pval <- cor.test(as.numeric(Ages), as.numeric(signenesNorm[i, ]), method = "spearman")$p.value
    SpearmanCorrelation <- c(SpearmanCorrelation, Scor)
    Pvalues <- c(Pvalues, Pval)
    HUM <- c(HUM, i)
    d <- data.frame(Days = c(as.numeric(Ages)), HumanT = c(as.numeric(signenesNorm[i,
        ])))
    colnames(d) = c("Days", i)
}

CorrTableSpearman <- data.frame(SigHuman_vsAge = c(HUM), Spearman = c(SpearmanCorrelation),
    Pvalue = c(Pvalues))
CorrTableSpearman$Padjusted <- p.adjust(CorrTableSpearman$Pvalue, method = "BH")
CorrTableSpearman <- CorrTableSpearman[order(CorrTableSpearman$Spearman, decreasing = T),
    ]

write.table(CorrTableSpearman, file = "outputs/SpearmanCorrelation_gestionalAge_SignificantGenes.txt",
    quote = F, col.names = NA, row.names = TRUE, sep = "\t")
```

GSEA

Load the required libraries

```r
library(ReactomePA)
```

Add entrez identifer, remove NA and order the vector by log2FC

```r
lfshrink$entrez <- mapIds(org.Hs.eg.db, keys = rownames(lfshrink), column = "ENTREZID",
    keytype = "SYMBOL")
dat <- lfshrink
# filter the dataframe to remove NA values
dat <- na.omit(dat)
genelist <- dat$log2FoldChange
names(genelist) <- dat$entrez
# Sort the genelist by log2 FC
genelist = sort(genelist, decreasing = TRUE)
```

Run the GSEA

```
y <- gsePathway(genelist, pvalueCutoff = 1, pAdjustMethod = "BH", verbose = FALSE,
    minGSSize = 5, maxGSSize = 800, seed = T, nPerm = 1e+05, exponent = 2)
outGSEA_Reactome <- as.data.frame(y)
```

Microbiome Taxonomy analysis

Read the data, remove virus and phages, also remove parasites

```
Counts_microbes <- read.table("datafiles/RawCounts_Microbe_Renamed.txt", header = T,
    sep = "\t", row.names = 1)
Species <- rownames(Counts_microbes)
viralSpecies <- grep("virus|phage ", Species, value = TRUE)

Counts_microbes <- Counts_microbes[which(!rownames(Counts_microbes) %in% viralSpecies),
    ]
Counts_microbes <- Counts_microbes[grep("Plasmodium vivax", rownames(Counts_microbes),
    value = T, invert = T), ]
```

Create the DESeq2 object

```
dds_microbes <- DESeqDataSetFromMatrix(countData = Counts_microbes, colData = metadata,
    design = ~Condition)
```

Filter to only keep species with atleast 1000 reads to remove noise, resulting in 129 species

```
dds_microbes <- dds_microbes[rowSums(counts(dds_microbes)) > 1000, ]
```

Running the differential expression, important we use poscounts normalization here!

```
dds_microbes <- DESeq(dds_microbes, betaPrior = FALSE, , parallel = TRUE, BPPARAM = MulticoreParam(4),
    sfType = "poscounts")
normCounts_microbes <- counts(dds_microbes, normalized = TRUE)

write.table(normCounts_microbes, file = "outputs/NormalizedCounts_Microbes.txt",
    col.names = NA, row.names = TRUE, sep = "\t", quote = FALSE, na = "NA")
```

The results from the Case vs control comparison is extracted while the foldchanges are shrunken using the normal method. The microbes are sorted by padjusted values.

```
lfshrink_microbes <- lfcShrink(dds_microbes, contrast = c("Condition", "Preterm",
    "Term"), type = "normal", parallel = TRUE, BPPARAM = MulticoreParam(4))
lfshrink_microbes <- lfshrink_microbes[order(lfshrink_microbes$padj), ]
write.table(lfshrink_microbes, file = "outputs/Microbes_Preterm_vs_Term.txt", col.names = NA,
    row.names = TRUE, sep = "\t", quote = FALSE, na = "NA")
```

Plots

Making the volcano plot with labels of the significant genes

```r
microbesOfInterest <- rownames(lfshrink_microbes[which(lfshrink_microbes$padj < 0.05),
    ])

plot(EnhancedVolcano(lfshrink_microbes, lab = rownames(lfshrink_microbes), x = "log2FoldChange",
    y = "padj", title = "Preterm vs Term", pCutoff = 0.05, FCcutoff = 2, selectLab = microbesOfInterest,
    legendIconSize = 2, drawConnectors = TRUE, widthConnectors = 0.5, colConnectors = "grey30",
    labSize = 5, ylim = c(0, 35), xlim = c(-5, 5), pointSize = c(ifelse(lfshrink_microbes$padj <
        0.05, 4, 2))))
```

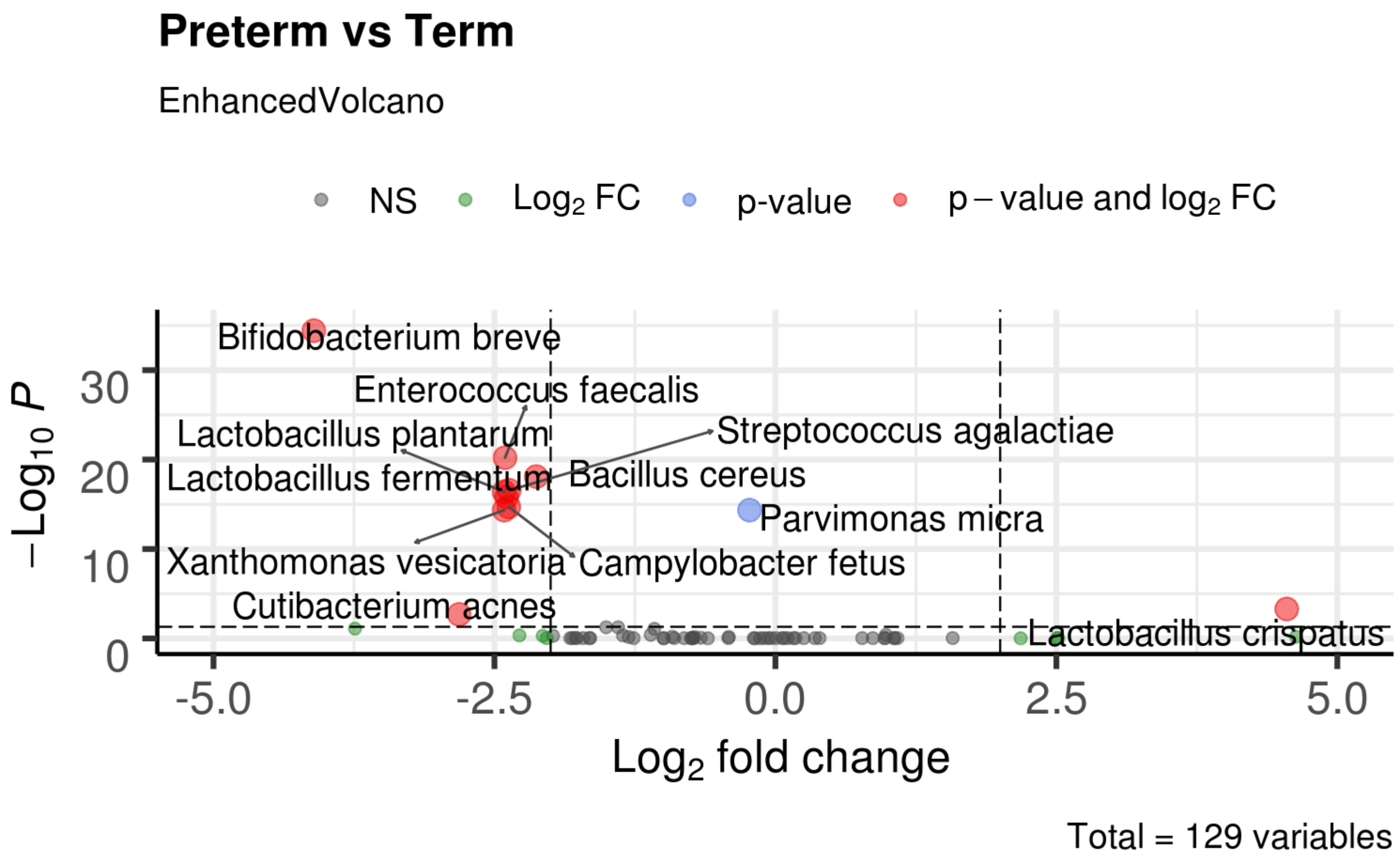

Creating the heatmap of the significant microbes

```r
signormCounts_microbes <- normCounts_microbes[microbesOfInterest, rownames(metadata[order(metadata$Condition,
    decreasing = T), , drop = FALSE])]

tsignormCounts_microbes <- t(signormCounts_microbes)
```

```r
breaksList = seq(0, 20, by = 0.1)
pheatmap.type(log2(tsignormCounts_microbes + 1), annRow = metadata, doTranspose = TRUE,
    show_colnames = FALSE, conditions = c("Term", "Preterm"), color = colorRampPalette(rev(brewer.pal(n = 7,
        name = "RdYlBu")))(length(breaksList)), breaks = breaksList, cellheight = 7,
    fontsize_row = 7)
```

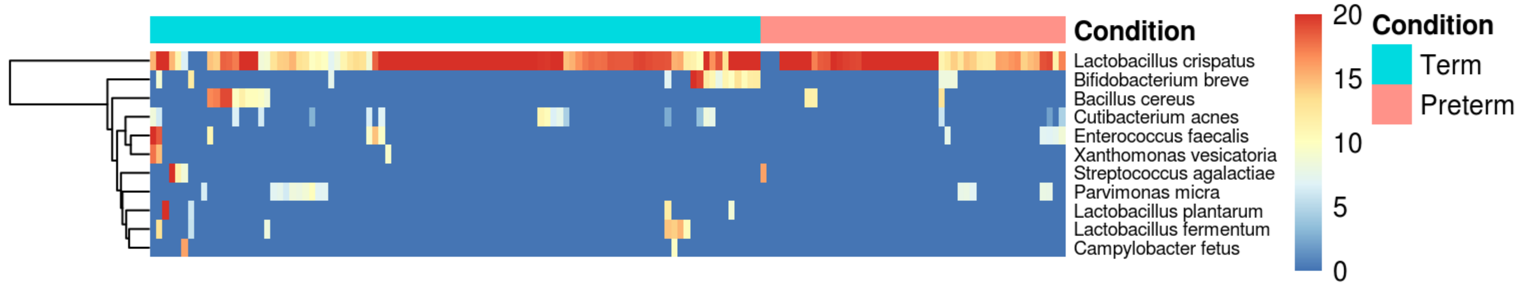

Creating the heatmap of the most common microbes

```r
mostcommonmicrobes <- c("Lactobacillus iners", "Lactobacillus crispatus", "Lactobacillus gasseri",
    "Gardnerella vaginalis", "Lactobacillus jensenii", "Lactobacillus paragasseri",
    "Bifidobacterium breve", "Aerococcus christensenii", "Campylobacter ureolyticus",
    "Sneathia amnii", "Ureaplasma parvum", "Lactobacillus sp. C25")

commonspeciesnorm <- normCounts_microbes[mostcommonmicrobes, rownames(metadata[order(metadata$Condition,
    decreasing = T), , drop = FALSE])]
tcommonspeciesnorm <- t(commonspeciesnorm)
```

```
breaksList = seq(0, 20, by = 0.1)
pheatmap.type(log2(tcommonspeciesnorm + 1), annRow = metadata, doTranspose = TRUE,
    show_colnames = FALSE, conditions = c("Term", "Preterm"), color = colorRampPalette(rev(brewer.pal(n = 7,
        name = "RdYlBu")))(length(breaksList)), breaks = breaksList, cellheight = 7,
    fontsize_row = 7)
```

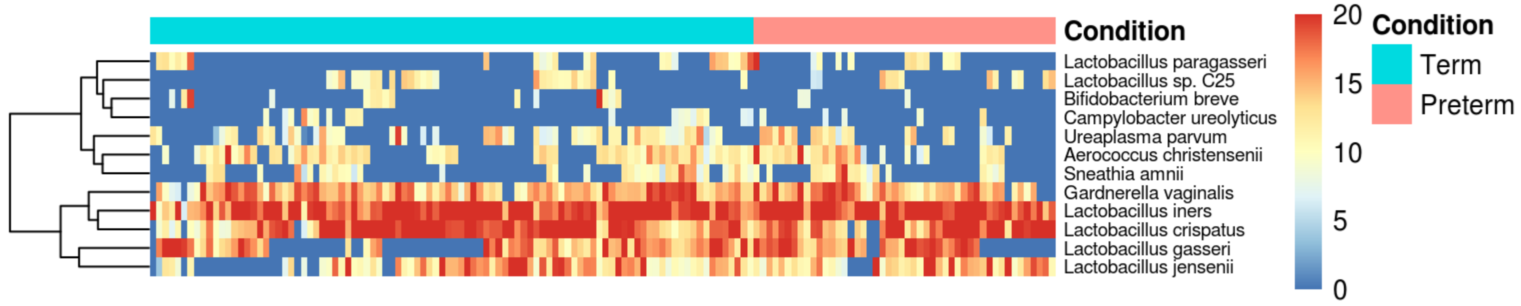

Microbiome reads vs human gene reads

Here we look at the distribution between reads from human reads and compared to annotated microbes

```
library(reshape2)

HumanSums <- as.data.frame(colSums(Counts))
MicrobiomeSums <- as.data.frame(colSums(Counts_microbes))
```

```r
sum_table <- merge(HumanSums, MicrobiomeSums, by = 0)
rownames(sum_table) <- sum_table$Row.names
sum_table$Row.names <- NULL
colnames(sum_table) <- c("HumanCountSum", "BacteriaCountSum")
humanfracs <- sum_table$HumanCountSum/(sum_table$HumanCountSum + sum_table$BacteriaCountSum)
names(humanfracs) <- rownames(sum_table)
humanfracs <- sort(humanfracs, decreasing = F)
orders <- rep(1:144)
orders <- cbind(as.data.frame(humanfracs), as.data.frame(orders))
orders$humanfracs <- NULL


ControlSamples <- rownames(metadata[metadata$Condition == "Term", , drop = FALSE])
CaseSamples <- rownames(metadata[metadata$Condition == "Preterm", , drop = FALSE])

datatable <- melt(as.matrix(sum_table))
datatable <- merge(datatable, orders, by.x = "Var1", by.y = 0)
datatable <- datatable[order(datatable$orders), ]

datatable_controls <- datatable[which(datatable$Var1 %in% ControlSamples), ]
datatable_controls <- datatable_controls[order(datatable_controls$orders), ]

ggplot(datatable_controls, aes(fill = Var2, y = value, x = reorder(Var1, orders))) +
    geom_bar(position = "fill", stat = "identity") + coord_polar() + theme_minimal() +
    xlab("") + ylab("") + theme(axis.title.y = element_blank(), axis.text.y = element_blank(),
    axis.text.x = element_blank(), panel.grid.major = element_blank(), panel.grid.minor = element_blank(),
    panel.background = element_blank(), legend.title = element_text(size = 20), legend.text = element_text(size = 20)) +
    ggtitle("Term") + labs(fill = "Group") + scale_fill_brewer(palette = "Dark2")
```

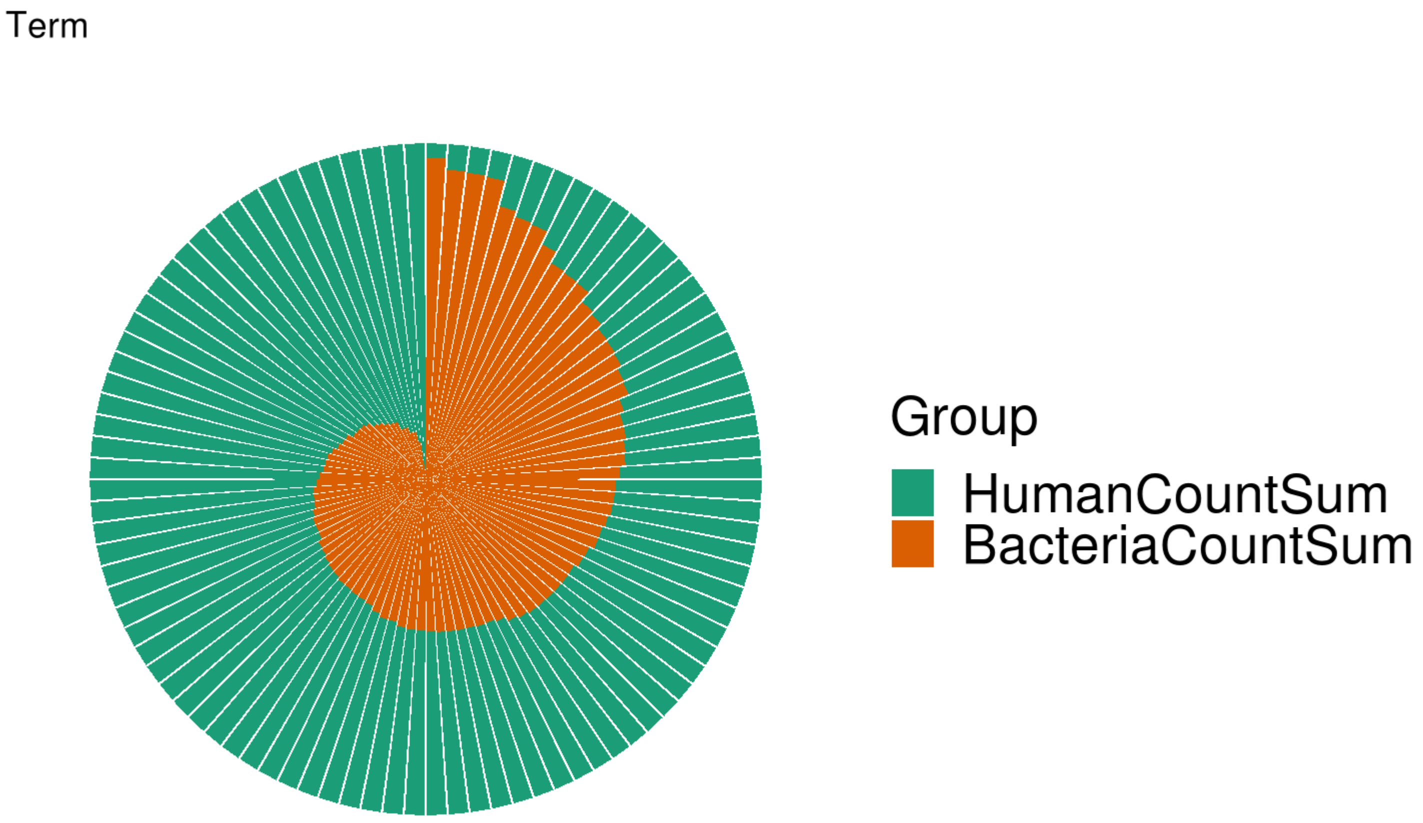


```r
datatable_Cases <- datatable[which(datatable$Var1 %in% CaseSamples), ]
datatable_Cases <- datatable_Cases[order(datatable_Cases$orders), ]
ggplot(datatable_Cases, aes(fill = Var2, y = value, x = reorder(Var1, orders))) +
    geom_bar(position = "fill", stat = "identity") + coord_polar() + theme_minimal() +
    xlab("") + ylab("") + theme(axis.title.y = element_blank(), axis.text.y = element_blank(),
    axis.text.x = element_blank(), panel.grid.major = element_blank(), panel.grid.minor = element_blank(),
    panel.background = element_blank(), legend.title = element_text(size = 20), legend.text = element_text(size = 20)) +
    ggtitle("Preterm") + labs(fill = "Group") + scale_fill_brewer(palette = "Dark2")
```

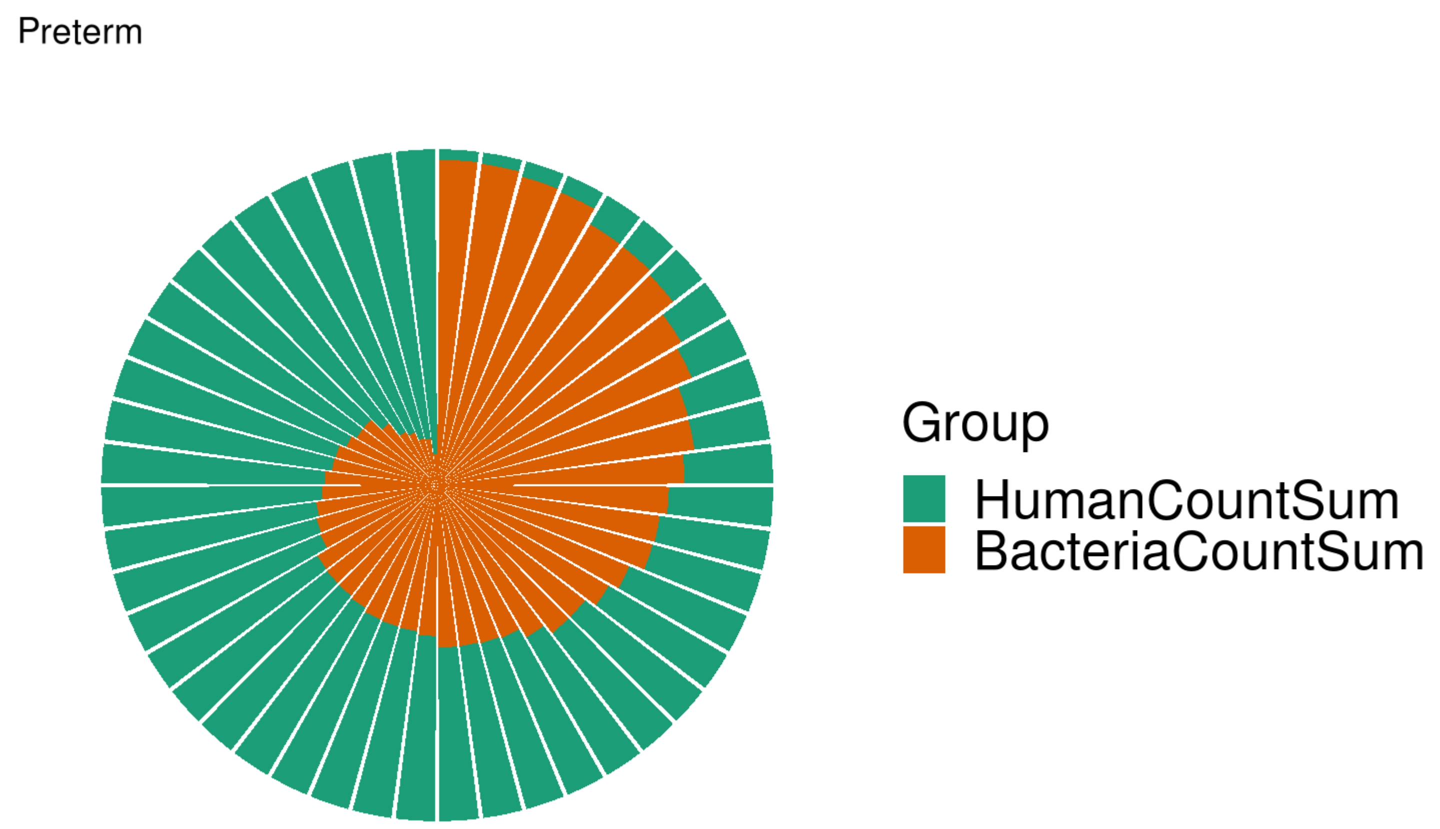

Most common species plot

Here we plot top 10 species in the Control group and in the Case group

```
library(ggplot2)
library(data.table)
library(forcats)
```

Plot the 10 most common species in case and control

```
gg_color_hue <- function(n) {
    hues = seq(15, 375, length = n + 1)
    hcl(h = hues, l = 65, c = 100)[1:n]
}
```

```r
CountTable_Controls <- Counts_microbes[, ControlSamples]
CountTable_Case <- Counts_microbes[, CaseSamples]

t <- data.frame(Controls = c(head(rownames(CountTable_Controls[order(rowSums(CountTable_Controls),
    decreasing = T), ]), n = 20)), Case = c(head(rownames(CountTable_Case[order(rowSums(CountTable_Case),
    decreasing = T), ]), n = 20)))

Control <- Counts_microbes[t$Controls[1:10], ControlSamples]
Control <- reshape2::melt(as.matrix(Control))
setDT(Control)[, `:=`(frac, value/sum(value)), by = Var2]

Case <- -Counts_microbes[t$Case[1:10], CaseSamples]
Case <- reshape2::melt(as.matrix(Case))
setDT(Case)[, `:=`(frac, value/sum(value)), by = Var2]
Top10CaseControls <- unique(c(t$Controls[1:10], t$Case[1:10]))
my_colors = gg_color_hue(length(Top10CaseControls))
names(my_colors) <- Top10CaseControls
Case$Var1 <- factor(Case$Var1, levels = Top10CaseControls)
Control$Var1 <- factor(Control$Var1, levels = Top10CaseControls)

ggplot(Control, aes(fill = Var1, x = reorder(Var2, frac, FUN = median), y = frac)) +
    geom_bar(stat = "identity") + ylim(-0.3, 1.01) + coord_polar() + theme_minimal() +
    xlab("") + ylab("") + theme(axis.title.y = element_blank(), axis.text.y = element_blank(),
    axis.text.x = element_blank(), panel.grid.major = element_blank(), panel.grid.minor = element_blank(),
    panel.background = element_blank(), legend.title = element_text(size = 20), legend.text = element_text(size = 20)) +
    ggtitle("Term") + labs(fill = "Species") + scale_fill_manual("Species", values = my_colors)
```

Term

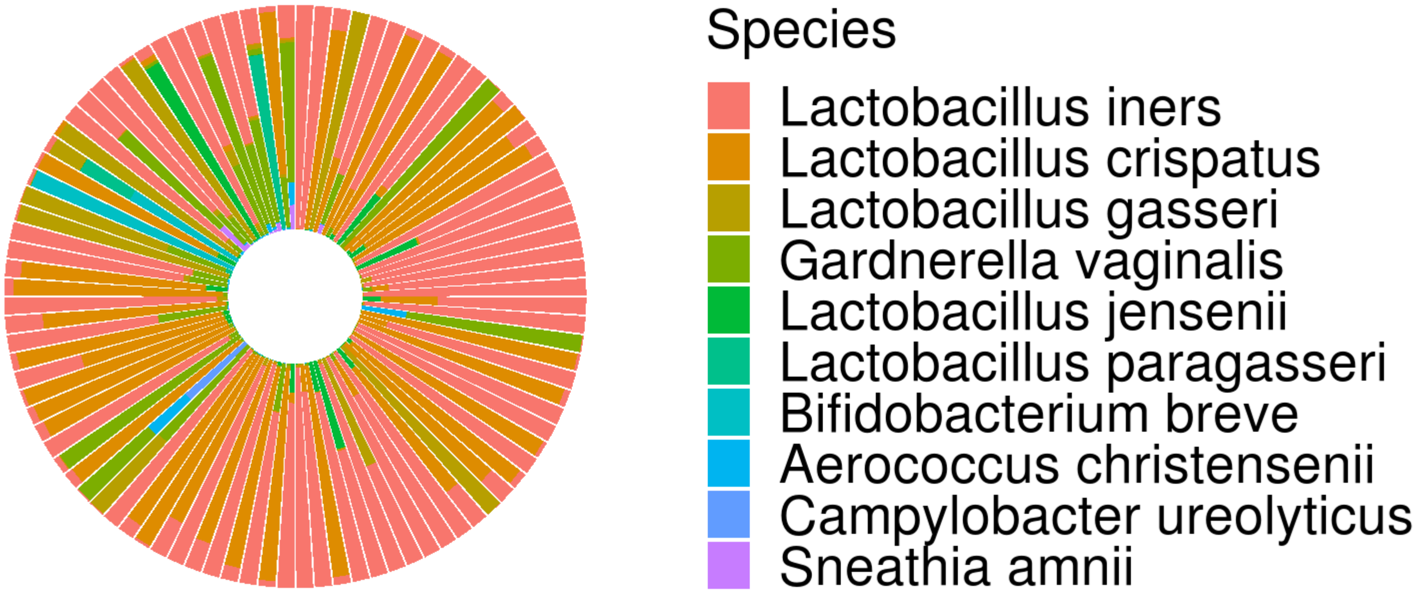


```
ggplot(Case, aes(fill = Var1, x = reorder(Var2, frac, FUN = median), y = frac)) +
    geom_bar(stat = "identity") + ylim(-0.3, 1.01) + coord_polar() + theme_minimal() +
    xlab("") + ylab("") + theme(axis.title.y = element_blank(), axis.text.y = element_blank(),
    axis.text.x = element_blank(), panel.grid.major = element_blank(), panel.grid.minor = element_blank(),
    panel.background = element_blank(), legend.title = element_text(size = 20), legend.text = element_text(size = 20)) +
    ggtitle("Preterm") + scale_fill_manual("Species", values = my_colors)
```

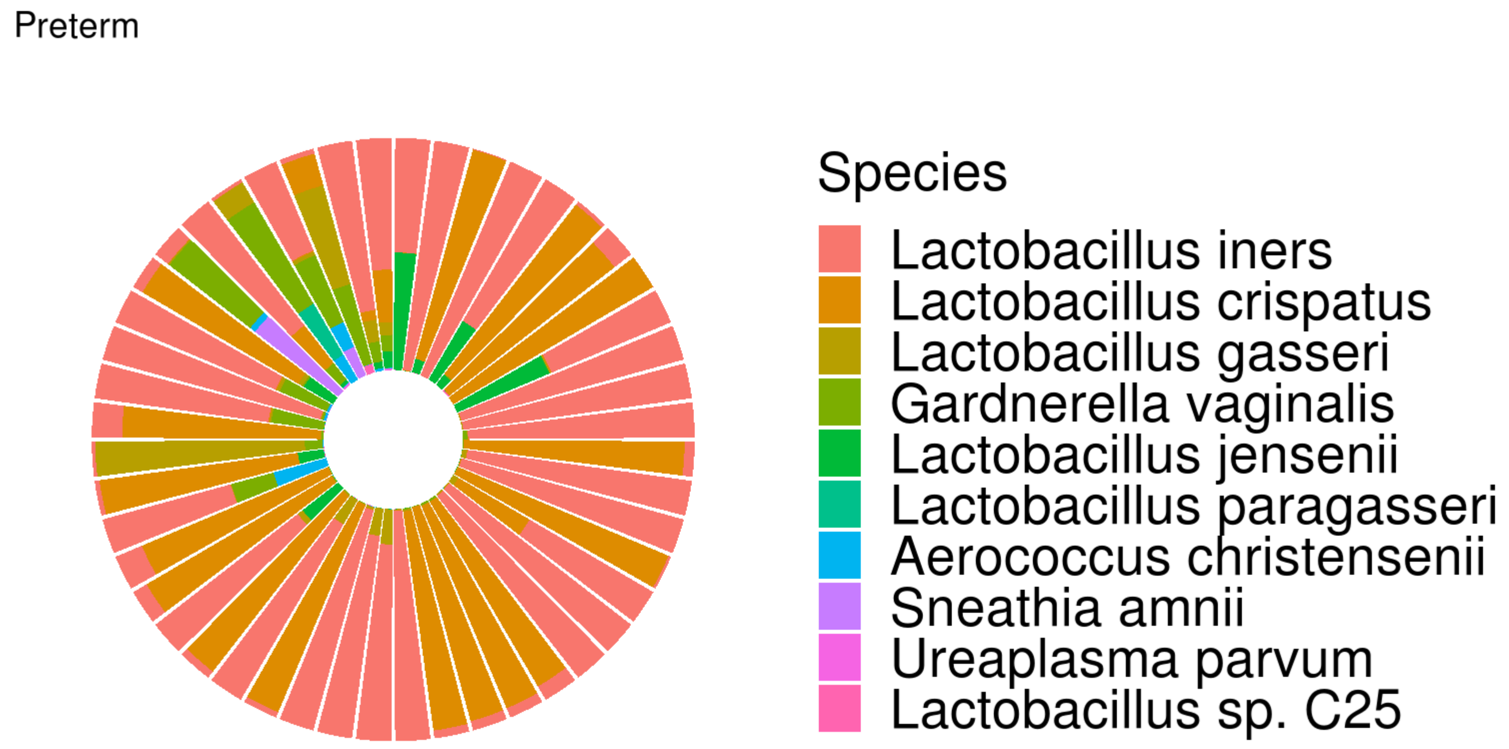

Bacterial Load plot (supplementary)

Bacterial load is looking at the amount of classified microbes compared to the total amount of classified material (human and microbes).

```
BacterialLoad <- read.table("datafiles/BacterialLoad_Renamed.txt", header = T, sep = "\t")
colnames(BacterialLoad) <- c("Row.names", "BacterialLoad", "Condition")
ordered <- BacterialLoad[order(BacterialLoad$Condition, BacterialLoad$BacterialLoad,
    decreasing = FALSE), ]$Row.names
BacterialLoad$Row.names <- factor(BacterialLoad$Row.names, levels = c(ordered))
ggplot(BacterialLoad, aes(fill = Condition, y = BacterialLoad, x = Row.names)) +
    geom_bar(stat = "identity") + ggtitle("Bacterial Load CaseControl") + theme(axis.text.x = element_blank()) +
    ylim(0, 1) + labs(x = "Sample")
```

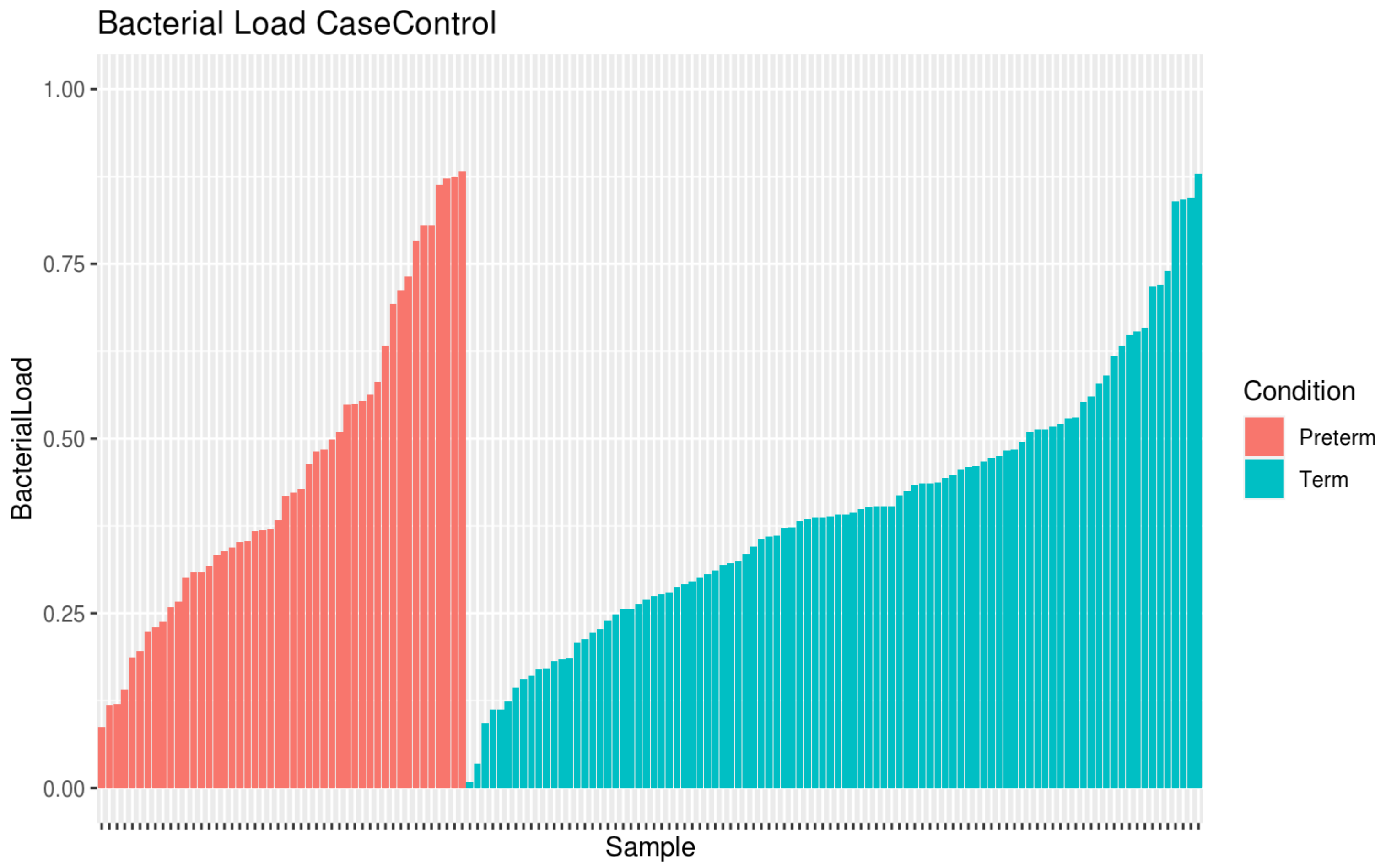
Bacterial Load CaseControl
1.00
0.75
0.50
0.25
0.00
BacterialLoad
Sample
Condition
Preterm
Term
